# GUEST: an R package for handling estimation of graphical structure and multiclassification for error-prone gene expression data

**DOI:** 10.1093/bioinformatics/btae731

**Published:** 2024-12-11

**Authors:** Li-Pang Chen, Hui-Shan Tsao

**Affiliations:** Department of Statistics, National Chengchi University, Taipei 116, Taiwan (R.O.C.); Department of Statistics, National Chengchi University, Taipei 116, Taiwan (R.O.C.)

## Abstract

**Summary:**

In bioinformatics studies, understanding the network structure of gene expression variables is one of the main interests. In the framework of data science, graphical models have been widely used to characterize the dependence structure among multivariate random variables. However, the gene expression data possibly suffer from ultrahigh-dimensionality and measurement error, which make the detection of network structure challenging and difficult. The other important application of gene expression variables is to provide information to classify subjects into various tumors or diseases. In supervised learning, while linear discriminant analysis is a commonly used approach, the conventional implementation is limited in precisely measured variables and computation of their inverse covariance matrix, which is known as the precision matrix. To tackle those challenges and provide a reliable estimation procedure for public use, we develop the R package GUEST, which is known as ***G***raphical models for ***U***ltrahigh-dimensional and ***E***rror-prone data by the boo***ST***ing algorithm. This R package aims to deal with measurement error effects in high-dimensional variables under various distributions and then applies the boosting algorithm to identify the network structure and estimate the precision matrix. When the precision matrix is estimated, it can be used to construct the linear discriminant function and improve the accuracy of the classification.

**Availability and implementation:**

The R package is available on https://cran.r-project.org/web/packages/GUEST/index.html.

## 1 Introduction

In bioinformatics studies, an important research interest lies on analysis of gene expression or RNA-sequencing variables, where the dimension of the variables *p* is usually larger than the sample size *n*. One of the important questions in gene expression data analysis is exploring network structures of gene expressions, which reflect pairwise dependence structure among gene expressions. To detect the network structure, graphical models have been useful tools in data science, whose key idea is to use a parametric probabilistic model to characterize random variables and then estimate the parameters that are used to characterize pairs of random variables. In the framework of graphical models, a large body of estimation methods have been developed, such as Banerjee *et al.* (2008), Cai *et al.* (2011a), Friedman *et al.* (2008), Ravikumar *et al.* (2010), and Yuan and Lin (2007). Other methods and more discussion can be found in Chen (2024).

However, ultrahigh-dimensionality, especially when *p* is extremely larger than *n*, is a challenging feature to detect the network structure. To address this challenge, some methods have been proposed, including Qiu *et al.* (2016), Wang *et al.* (2020), and Yang *et al.* (2023), but those approaches primarily focused on continuous variables but may not be applicable to discrete variables. In applications, gene expressions may suffer from measurement errors, which are due to imprecise measurements or wrong records. It is well known that ignoring measurement error effects would induce biases in the estimation, yielding that analysis results are unreliable, counterintuitive, or different from what the researchers expect. To address this challenge, Chen and Yi (2022) and Wainwright (2019, p.369) proposed valid methods to correct for measurement error effects. However, they might be invalid to tackle ultrahigh-dimensional settings. In the presence of those two complex features, rare methods have been available to handle them simultaneously.

In addition to the detection of the network structure, classification of various cancers/tumors by using gene expressions is also a challenging topic. In supervised learning, linear discriminant analysis (LDA) is one of the popular approaches (e.g. Witten and Tibshirani 2011) to deal with classification because of its flexibility and easy implementation. A key element in the linear discriminant function is the inverse of the covariance matrix, which is also known as the precision matrix. The conventional implementation is to estimate the precision matrix by the inverse of the empirical estimate of the covariance matrix. In the presence of high-dimensionality, however, the covariance matrix is singular and is not invertiable. Moreover, as noted by Cai *et al.* (2013), Chen and Yi (2022), James *et al.* (2010), and Leclerc (2008), gene regulatory networks are usually sparse, indicating that rare edges connect pairs of genes. In graphical model theory, as shown in Appendix B1 of the Supplementary Material, when genes are taken as variables, the parameters for pairs of genes are entries in the precision matrix, and zero (or nonzero) values of the parameters reflect independence (or dependence) of two genes. Consequently, sparse gene regulatory networks induce many unconnected edges and zero values of parameters for pairs of genes, yielding sparse precision matrices. Due to this natural phenomenon, it seems to be unrealistic to implement the empirical estimate of the covariance matrix directly. To tackle this issue, one may estimate the precision matrix by using the graphical model techniques and then implement the estimator to the linear discriminant function (e.g. Chen 2022a). However, this approach may not be valid when *p* is extremely larger than *n* or gene expressions suffer from measurement error.

In the framework of graphical model theory or supervised learning in classification, several R packages have been developed for practical implementation. As summarized in Table A1 placed in the Supplementary Material, all existing R packages in the current development do not take measurement error correction into account, and rare R packages are valid to address high-dimensional data. In addition, the implementation of existing R packages is not valid to handle measurement error effects when doing classification.

**Table 1.** Classification results for small round blue cell tumors gene expression data.

Criteria	glasso	GUEST					huge	space	QUIC	RLDA
		0.15	0.35	0.55	0.75	0.95				
PRE_1_	0.963	1.000	1.000	1.000	1.000	1.000	1.000	0.000	1.000	0.000
PRE_3_	1.000	1.000	1.000	1.000	1.000	1.000	0.917	0.121	0.917	0.059
PRE_3_	0.947	0.857	0.857	0.947	0.900	0.900	0.900	0.000	0.947	0.417
PRE_4_	0.923	1.000	1.000	1.000	1.000	1.000	1.000	0.000	1.000	0.000
PRE	0.958	0.964	0.964	0.987	0.966	0.966	0.931	0.000	0.966	0.000
REC_1_	0.897	0.966	0.966	0.966	1.000	1.000	1.000	0.364	1.000	0.273
REC_2_	1.000	1.000	1.000	1.000	1.000	1.000	1.000	0.000	1.000	0.556
REC_3_	1.000	1.000	1.000	1.000	0.960	0.960	0.960	0.000	0.960	0.000
REC_4_	0.960	0.920	0.920	1.000	0.975	0.975	0.954	0.030	0.966	0.119
REC	0.964	0.972	0.972	0.992	0.981	0.981	0.973	0.091	0.981	0.207
F	0.961	0.968	0.968	0.989	0.978	0.978	0.963	0.045	0.974	0.151

Consequently, to tackle challenges of complex features and unify the estimation of the network structure and classification in the same R package for public use, we develop a new R package called GUEST (Tsao and Chen 2024, https://cran.r-project.org/package=GUEST). This R package contains two parts: first, the estimation of the network structure is provided. Our estimation procedure can correct for measurement errors in continuous, binary, or count data. In addition, we adopt the feature screening technique and the boosting algorithm to efficiently detect the informative pairwise dependence and estimate the precision matrix. Second, we modify the linear discriminant function with measurement error corrected. With an estimated precision matrix equipped, we can do classification and improve the performance of existing approaches.

## 2 Overview of the R package GUEST

There are two main functions in this R package, which are primarily used to detect the network structure for high-dimensional random variables and implement the linear discriminant function for the classification, respectively. Both functions in this R package are designed to handle measurement error effects. In addition, since the parameters in measurement error models are usually unknown, both functions in the R package GUEST provide arguments for the specification of parameters in measurement error models, so that users are flexible to implement values that they wish to examine or specific values from their background knowledge of the datasets. Moreover, if one believes that the dataset has no measurement error, then those arguments can be specified as zero values, implying that our R package can also be implemented to a dataset without measurement error in variables.

### 2.1 boost.graph

The first function boost.graph is used to detect the network structure for exponential family distributed random variables, including continuous, binary, or count data, with measurement error corrected. The operations of this function contain several key steps: first, regression calibration (e.g. Chen and Yi 2021) is implemented to correct for measurement error effects. With the corrected variables obtained by regression calibration, the second step takes the Chatterjee’s measure (Chatterjee 2021) to detect highly dependent pairs of random variables and remove independent or weakly dependent ones. As discussed in Chen (2023), who considered ultrahigh-dimensional regression models, the feature screening procedure based on the Chatterjee’s measure has the sure screening property, ensuring that the set containing retained variables covers the set of truly informative variables with a large probability. Unlike the existing literature, our approach here is to retain dependent pairs of random variables for the network structure. Finally, the last step is to combine the boosting algorithm with the neighborhood selection method (e.g. Chen and Yi 2022) to detect dependent pairs of random variables and estimate the associated parameters; a detailed procedure can be found in Algorithm 1 of the Supplementary Material.

Unlike other existing R packages (e.g. Cai *et al.* 2011b, https://CRAN.R-project.org/package=clime; Friedman *et al.* 2019, https://CRAN.R-project.org/package=glasso; Wan *et al.* 2016), this function in the R package GUEST is valid to handle ultrahigh-dimensional random variables and correctly detect network structures for random variables. The other key contribution is that the R package GUEST is useful for dealing with measurement error effects for normal or non-normal random variables, indicating that the R package is expected to analyze gene expression or RNA-sequencing data. Simulation results in the Supplementary Material also reveal that the R package GUEST generally outperforms some commonly used R packages in the literature. Regarding the methodology, our main implementation is the boosting procedure, which enables us to detect pairwise dependence and derive the estimator iteratively. The main advantage of this implementation is that one simply adopts finite iterations to obtain the desired results instead of using the conventional regularization approaches (e.g. Friedman *et al.* 2008, Ravikumar *et al.* 2010). Moreover, the function boost.graph provides flexible arguments for users to specify parameters in the algorithm or in measurement error models. The outputs of this function include the estimator and pairs obtained by the feature screening procedure. For visualization, we also display the estimated network structure.

### 2.2 LDA.boost

The second function LDA.boost is used to implement the linear discriminant function for the classification with C≥2 multi-labels, which is given by

(1) $${\hat{\delta }}_{c}^{*}(\hat{X})≜\;\text{log}\;\left({\hat{\pi }}_{c}\right)-\frac{1}{2}{\hat{\mu }}_{c}^{*⊤}\hat{\Theta }{\hat{\mu }}_{c}^{*}+{\hat{X}}^{⊤}\hat{\Theta }{\hat{\mu }}_{c}^{*}$$

for c=1,…,C, where $\hat{X}$ is the corrected variables obtained by regression calibration, ${\hat{\pi }}_{c}$ is the proportion of subjects in the class *c*, and ${\hat{\mu }}_{c}^{*}$ is the vector of the empirical mean of $\hat{X}$ under the class *c*. The crucial issue is the implementation of $\hat{\Theta }$. In conventional LDA, $\hat{\Theta }$ is usually taken as the inverse of the estimated covariance matrix of the covariates. However, a covariance matrix could be singular if the dimension of variables were extremely greater than the sample size. In addition, the conventional implementation may possibly ignore the network structure, or the sparsity of $\hat{\Theta }$, which may affect the performance of the classification. Alternatively, we suggest implementing the $\hat{\Theta }$ estimated by methods in graphical model theory, so that the network structure can be taken into account. Consequently, the function LDA.boost provides a flexible argument for the implementation of $\hat{\Theta }$. Our simulation studies in the Supplementary Material examine various implementations obtained by the R packages glasso, clime, huge, space, QUIC, and GUEST. Numerical results suggest that our R package generally gives the satisfactory performance of the classification.

## 3 Application and findings

We apply the R package GUEST to analyze the small round blue cell tumors (SRBCT) gene expression data that were described in Khan *et al.* (2001). The SRBCT dataset includes four similar childhood tumors: Ewing sarcoma (EWS), Burkitt lymphoma (BL), neuroblastoma (NB), and rhabdomyosarcoma (RMS), and are labeled 1, 2, 3, and 4, respectively. The sample size is *n* = 83, which is divided by $n_{1}=29$ in EWS, $n_{2}=11$ in BL, $n_{3}=18$ in NB, and $n_{4}=25$ in RMS. In addition, there are *p* = 2308 gene expression values, whose names can be found on the website.

The first target aims to detect the network structure of gene expressions. As noted by Chen and Yi (2022), gene expressions are possibly contaminated with measurement error. Since gene expression values in this dataset are continuous, we use the classical measurement error model with the covariance matrix $\Sigma_{\epsilon }$ treated as the noise term to characterize measurement error in variables, where $\Sigma_{\epsilon }$ is used to show the magnitudes of measurement error effects and reflect the difference between observed and unobserved variables; more detailed formulations can be found in Appendix B2 of the Supplementary Material. Since $\Sigma_{\epsilon }$ is unknown in applications and there is no auxiliary information to estimate it, to implement the function boost.graph, we primarily conduct sensitivity analyses by specifying $\Sigma_{\epsilon }$ as a diagonal matrix with common entries $\sigma_{\epsilon }=0.15,0.35,0.55,0.75$, or 0.95. In addition, we also examine regularized LDA (RLDA, Mahadi *et al.* 2024) and the existing R packages glasso, huge, space, and QUIC, which do not take measurement error correction into account.

**Figure 1.**
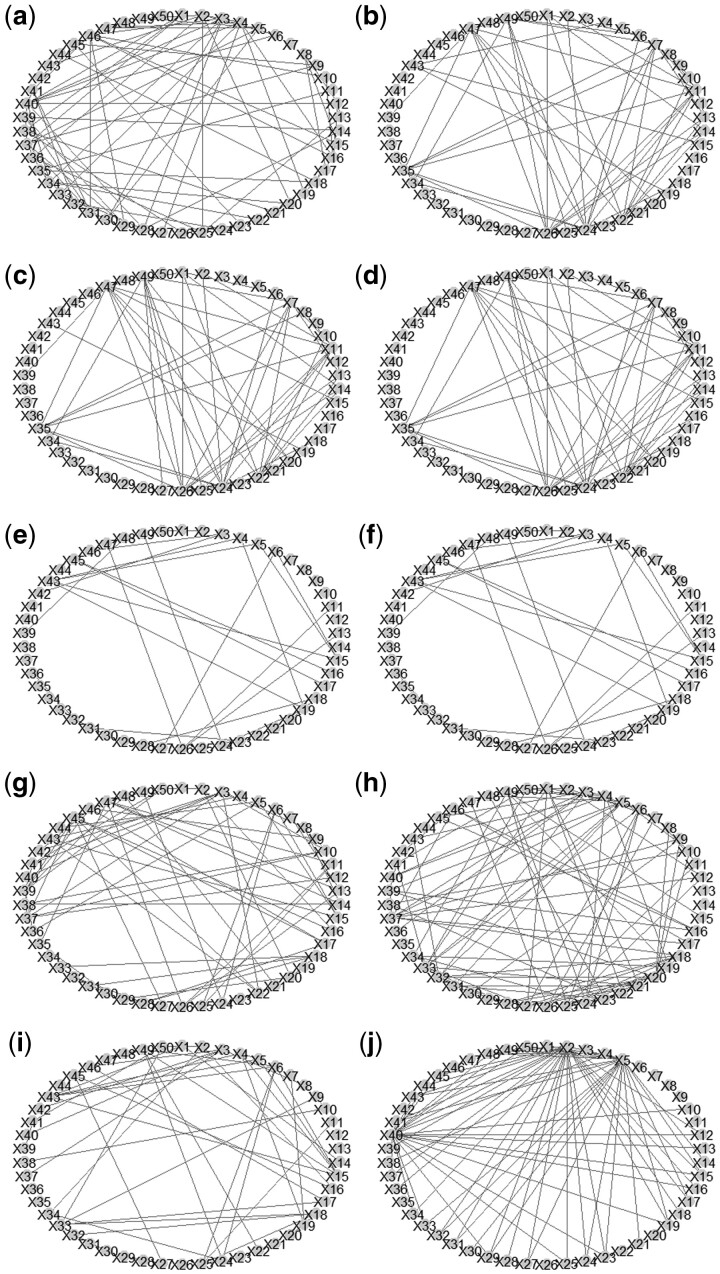
The visualization of estimated network structures of the SRBCT data. Graph (a) is derived by glasso; Graphs (b)–(f) are derived by GUEST with $\sigma_{\epsilon }$ being 0.15, 0.35, 0.55, 0.75, and 0.95, respectively. Graphs (g), (h), and (i) are determined by the R packages huge, space, and QUIC, respectively. Graph (j) is derived by RLDA.

To clearly demonstrate the estimated graphs and to facilitate discussion, we simply display the first 50 variables *X*_1_–*X*_50_ in Fig. 1. We can see that there are some edges that are detected by two R packages glasso and GUEST, such as $\left(X_{47},X_{11}\right)$ and $\left(X_{47},X_{26}\right)$, where *X*_47_, *X*_11_, *X*_26_ are “heterogeneous nuclear ribonucleoprotein C”, “large ribosomal protein P0”, and “Human 90-kDa heat-shock protein gene”, respectively; a pair $\left(X_{49},X_{25}\right)$ is only detected by the R packages GUEST and space, where *X*_49_ and *X*_25_ are “lactate dehydrogenase A” and “calmodulin 2”, respectively. Moreover, it is also notable to see that some pairs are uniquely detected by the R package GUEST. For example, when $\sigma_{\epsilon }=0.15,0.35$, or 0.55, pairs $\left(X_{49},X_{26}),\;(X_{35},X_{7}),\;(X_{35},X_{8}\right)$, and $\left(X_{35},X_{11}\right)$ are identified, where *X*_35_, *X*_7_, and *X*_8_ represent “adenine nucleotide translocator 3 (liver)”, “guanine nucleotide binding protein (G protein)”, and “pre-mRNA splicing factor SF3a (120 kDa subunit)”, respectively. On the other hand, when $\sigma_{\epsilon }=0.75$ or 0.95, a pair $\left(X_{31},X_{23}\right)$ is detected, where *X*_31_ and *X*_23_ are “glycogen synthase kinase 3 beta” and “glutamic-oxaloacetic transaminase 1”, respectively. The detection of pairwise interactions seems to be affected by various magnitudes of measurement error effects.

The second target aims to handle multi-label classification. We implement the linear discriminant function [Equation (1)] with the estimate $\hat{\Theta }$ derived by the RLDA method and R packages glasso, huge, space, QUIC, and GUEST accommodated. For a subject *i* with i=1,…,n, we denote ${\hat{Y}}_{i}\in \left\{1,2,3,4\right\}$ as the predicted class label determined by the linear discriminant function, and denote $Y_{i}\in \left\{1,2,3,4\right\}$ as the true class label with *Y_i_* = 1 representing that the patient *i* in in EWS, *Y_i_* = 2 reflecting that the patient *i* belongs to BL, *Y_i_* = 3 reflecting that the patient *i* is in NB, and *Y_i_* = 4 reflecting that the patient *i* is in RMS. To assess the performance of the classification, we examine Precision (PRE), Recall (REC), and F-values (e.g. Chen 2022a). Specifically, for the *k*th class with k=1,2,3,4, the true positive (TP), the false positive (FP), and the false negative (FN) are respectively defined as ${\text{TP}}_{k}=\sum_{i=1}^{n}I(Y_{i}=k,{\hat{Y}}_{i}=k),\;{\text{FP}}_{k}=\sum_{i=1}^{n}I(Y_{i}\neq k,{\hat{Y}}_{i}=k)$, and ${\text{FN}}_{k}=\sum_{i=1}^{n}I(Y_{i}=k,{\hat{Y}}_{i}\neq k)$.

After that, values of precision and recall for the *k*th class with k=1,2,3,4 are respectively given by ${\text{PRE}}_{k}=\frac{{\text{TP}}_{k}}{{\text{TP}}_{k}+{\text{FP}}_{k}}$ and ${\text{REC}}_{k}=\frac{{\text{TP}}_{k}}{{\text{TP}}_{k}+{\text{FN}}_{k}}$, which yield the values of overall precision and recall:

(2) $$\text{PRE}=\frac{1}{4}\sum_{k=1}^{4}{\text{PRE}}_{k}\;\text{and}\;\text{REC}=\frac{1}{4}\sum_{k=1}^{4}{\text{REC}}_{k}.$$

By Equation (2), the F-value is defined as

(3) $$F=2\;\frac{\text{PRE}\times \text{REC}}{\text{PRE}+\text{REC}}.$$

As noted in Chen (2022a,b), higher values of Equations (2) and (3) reflect more accurate classification.

Numerical results are summarized in Table 1. We find that the R package GUEST under various $\sigma_{\epsilon }$ generally outperforms the results derived by the RLDA method and other R packages. Specifically, it is clear to see that our method provides accurate classification with higher values of criteria [Equations (2) and (3)] except for precision for NB and recall for RMS. Moreover, based on sensitivity analyses, we find that the R package GUEST induces the most accurate classification result when $\sigma_{\epsilon }=0.55$, suggesting that the magnitude of measurement error is about $\sigma_{\epsilon }=0.55$, and implementing 0.15,0.35 or 0.75, 0.95 may “underestimate” or “overestimate” $\sigma_{\epsilon }$.

In contrast, the implementation of existing approaches shows the impacts of measurement error and high dimensionality. In particular, the RLDA method, which ignores the feature of sparse network structure, has the worst classification performance. It may show that gene expressions in the SRBCT data might have the network structure, which affects the classification result.

## 4 Summary

In this article, we introduce a new R package GUEST, whose two equipped functions enable users to address detection of the network structure and the classification in the presence of ultrahigh-dimensional error-prone data. Both functions in the R package GUEST are novel to deal with complex data structures, and their arguments are flexible for users to handle various scenarios.

## Supplementary Material

btae731_Supplementary_Data

## Data Availability

A dataset analyzed in this project is publicly available at https://CRAN.R-project.org/package=plsgenomics.
