# Supplementary material for: GUEST: an R package for handling estimation of graphical structure and multiclassification for error-prone gene expression data
Source: Bioinformatics. 2024 Dec 11;40(12):btae731. doi: 10.1093/bioinformatics/btae731 (PMC11655624; doi:10.1093/bioinformatics/btae731)
Supplement: btae731_Supplementary_Data [file btae731_supplementary_data.pdf]

December 11, 2024

Supplementary Materials for "GUEST: An R package for handling estimation of graphical structure and multi-classification for error-prone gene expression data"

Li-Pang Chen*,[1] and Hui-Shan Tsao[2]

Department of Statistics, National Chengchi University, Taipei, Taiwan (R.O.C.)

[1] email: lchen723@nccu.edu.tw [2] email: 111354028@nccu.edu.tw

*The corresponding author

Abstract

Background: In bioinformatics studies, understanding the network structure of gene expression variables is one of main interests. In the framework of data science, graphical models have been widely used to characterize the dependence structure among multivariate random variables. However, the gene expression data possibly suffer from ultrahigh-dimensionality and measurement error. Those ubiquitous features may make the detection of network structure challenging and difficult. The other important application of gene expression variables is to provide information to classify subjects into various tumors or diseases. In supervised learning, while linear discriminant analysis is a commonly used approach, the conventional implementation is limited in precisely measured variables and computation of their inverse covariance matrix, which is known as the precision matrix.

Results: To tackle those challenges and provide a reliable estimation procedure for public use, we develop the R package `GUEST`, which is known as **G**raphical models for **U**ltrahigh-dimensional and **E**rror-prone data by the boo**ST**ing algorithm.

Conclusions: This package aims to deal with measurement error effects in high-dimensional variables under various distributions, and then applies the boosting algorithm to identify the network structure and estimate the precision matrix. When the precision matrix is estimated, it can be used to construct the linear discriminant function and improve the accuracy of the classification.

Keywords: Gene expression; measurement error; multi-label tumors; precision matrix; ultrahigh-dimensionality; variable selection.

A Background

In bioinformatics studies, a dataset usually contains gene expression or RNA-sequencing variables, and its size is usually larger than the sample size. One of important questions in analysis of gene expression data is to explore network structures of gene expressions, which reflect pairwise dependence structure among gene expressions. To detect the network structure, graphical models have been useful tools in data science, whose key idea is to use a parametric probabilistic model to characterize random variables, and then estimate the parameters that are used to characterize pairs of random variables. In the framework of graphical models, a large body of estimation methods have been developed, such as [2], [3], [16], [33], and [40]. Other methods and more discussions can be found in [8].

However, a challenge in gene expression data analysis is ultrahigh-dimensionality, especially when the dimension of variables is larger than the sample size. To deal with this challenge, some methods have been proposed, including [32, 37, 39], but those approaches primarily focused on continuous variables. In applications, gene expressions may suffer from measurement errors, which are due to imprecise measurements or wrong records. As pointed out by [6], ignoring measurement error effects would induce biases in the estimation, yielding that analysis results are unreliable, counter-intuitive, or different from what the researchers expect. To address this challenge, [12] and [35] (p.369) proposed valid methods to correct for measurement error effects. While those methods can address complex features, they may not be able to handle both challenges simultaneously.

In addition to the detection of the network structure, classification of various cancers by using gene expressions is also a challenging topic in bioinformatics. In supervised learning, linear discriminant analysis (LDA) is one of popular approaches (e.g., [38]) to deal with classification because of its flexibility and easy implementation. A key element in the linear discriminant function is the inverse of the covariance matrix, which is also known as the precision matrix. The conventional implementation is to estimate the precision matrix by the inverse of the empirical estimate of the covariance matrix. In the presence of high-dimensionality, however, the covariance matrix is singular and is not invertiable. Moreover, as noted by [4]; [12]; [21]; [26], gene regulatory networks are usually sparse, which indicate that rare edges connect pairs of genes. In graphical model theory, as shown in Section B.1 of the supplementary material, when genes are taken as variables, the parameters for pairs of genes are entries in the precision matrix, and zero (or nonzero) values of the parameters reflect independence (or dependence) of two genes. Consequently, sparse gene regulatory networks induce many unconnected edges and zero values of the parameters for pairs of genes, yielding sparse precision matrices. Due to this natural phenomenon, it seems to be unrealistic to implement the empirical estimate

of the covariance matrix directly. To tackle this issue, one may estimate the precision matrix by using the graphical model techniques, and then implement the estimator to the linear discriminant function (e.g., [10]). However, this approach may not hold when the dimension is extremely larger than the sample size or the gene expressions suffer from measurement error.

In the framework of graphical model theory or supervised learning in classification, several R packages have been developed for practical implementation. Some well known packages are listed in Table A1. We can find that all packages do not take measurement error correction into account, and rare packages are valid to address high-dimensional data. In the presence of various distributions in variables, three packages `XMRF`, `gRim`, and `mgm` are designed to deal with non-normal distributions. However, most packages have been archived in the latest R software version.

[Table A1 is about here]

Consequently, to tackle challenges of complex features and unify estimation of network structure and classification at the same package for public use, we develop a new R package called `GUEST`. This package contains two parts: first, the estimation of network structure is provided. Our estimation procedure can correct for measurement error in continuous, binary, or count data. In addition, we adopt the feature screening technique and the boosting algorithm to efficiently detect informative pairwise dependence and estimate the precision matrix. Second, we modify the linear discriminant function with measurement error corrected. With estimated precision matrix equipped, we can do classification and improve the performance from existing approaches.

The remainder is organized as follows. In the Section B, we introduce the data structure and relevant regression models. In Section C, we outline the estimation steps and the algorithm to derive the estimator. In Section D, we introduce the R package `GUEST`, including the functions, the arguments, and the outputs. In Section E, we apply the developed and existing packages to analyze a gene expression dataset. In Section F, we further conduct simulation studies to assess the performance of the method in the R packages. Finally, a general discussion is summarized in Section G.

B Data Structure and Regression Models

B.1 Graphical Models

Let $\mathbf{X} \triangleq (X_1, \cdots, X_p)^\top$ denote the p-dimensional random vector with $\mathbf{\Sigma} \triangleq \text{var}(\mathbf{X})$. Let n be the sample size, and let $\{\mathbf{X}_i : i = 1, \cdots, n\}$ denote the independent sample that has the same

distribution as $\mathbf{X}$, and denote X_{ir} as the rth component in $\mathbf{X}_i$. In our study, we allow the dimension p to be characterized as $p = \exp\{O(n^\kappa)\}$ for some positive constant κ, which is also known as $p \gg n$.

In graph theory, a graph is formed by $\mathcal{G} = (\mathcal{V}, \mathcal{E})$, where $\mathcal{V}$ is the set of vertices and $\mathcal{E}$ is the set of edges, or is known as the subset of $\mathcal{V} \times \mathcal{V}$. In the statistical perspective, the set $\mathcal{V}$ reflects the random variables, and pairs in the set $\mathcal{E}$ show the dependence of two random variables. Given the variables and the vertex set $\mathcal{V}$, the goal is to determine the edge set $\mathcal{E}$ and estimate the network structure of variables.

To characterize $\mathbf{X}$, we adopt the probabilistic model. In this study, we consider the following exponential family model

$$\mathbb{P}_{\boldsymbol{\Theta}}(\mathbf{X}) = \exp\left\{ \sum_{(k,l)\in E} \theta_{kl} \mathfrak{B}_k(X_k)\mathfrak{B}_l(X_l) + \sum_{r\in V} \mathfrak{C}_r(X_r) - \mathfrak{A}(\boldsymbol{\beta}, \boldsymbol{\Theta}) \right\}, \tag{B1}$$

where $\boldsymbol{\Theta} = [\theta_{kl}]$ is the $p \times p$ sparse matrix, in the sense that most entries θ_{kl} are zero; $\mathfrak{B}_r(\cdot)$ and $\mathfrak{C}_r(\cdot)$ are functions of the random variable X_r; and the function $\mathfrak{A}(\boldsymbol{\beta}, \boldsymbol{\Theta})$ is normalizing constant that ensures (B1) to be integrated as 1. As displayed in Figure B1, noting that θ_{kl} is taken as the coefficient of the pairwise interaction $\mathfrak{B}_k(X_k)\mathfrak{B}_l(X_l)$, it reflects that zero (or nonzero) value of θ_{kl} indicates independence (or dependence) between X_k and X_l, given other vertices $\mathcal{V} \setminus \{k, l\}$. As a result, to detect the network structure, it suffices to do variable selection and estimation for θ_{kl} for all $k \neq l$.

[Figure B1 is about here]

The other notable feature in (B1) is that the specification of two functions $\mathfrak{B}_k(\cdot)$ and $\mathfrak{C}_k(\cdot)$ reflects some well known graphical models. For example, specifying $\mathfrak{B}_r(X) = \frac{X}{\sigma}$ and $\mathfrak{C}_r(X) = -\frac{X^2}{2\sigma^2}$ with a known constant σ^2 for all $r \in \mathcal{V}$ and $X \in \mathbb{R}$ gives the Gaussian graphical model (GGM) [16]; if $\mathfrak{B}_r(X) = X$ and $\mathfrak{C}_r(X) = 0$ for all $r \in \mathcal{V}$ and $X \in \{-1, 1\}$, then (B1) reduces to the Ising model (ISM) [33]. More special cases can be found in [8]. To ease of discussion, we follow [12] to specify $\mathfrak{B}_r(X) = X$ for all $r \in \mathcal{V}$.

B.2 Measurement Error Models

In applications, the variable $\mathbf{X}$ is possibly subject to measurement errors, which are caused by wrong records or imprecise measurements. In other words, $\mathbf{X}$ can be unobserved variable, and the observed variable is denoted as $\mathbf{X}^* \triangleq (X_1^*, \cdots, X_p^*)^\top$, which is also known as the surrogate version of $\mathbf{X}$. Moreover, let $\{\mathbf{X}_i^* : i = 1, \cdots, n\}$ denote the independent and identically distributed sample of $\mathbf{X}^*$ with X_{ir}^* being the rth component in $\mathbf{X}_i^*$.

In the presence of measurement error, $\mathbf{X}^*$ cannot fully reflect $\mathbf{X}$, instead, there exist nonzero components $\mathbf{A}$ and $\mathbf{B}$, such that

$$\mathbf{X}^* = \mathbf{A}\mathbf{X} + \mathbf{B}, \tag{B2}$$

where $\mathbf{A}$ and $\mathbf{B}$ can be fixed or random effects. Due to different types of distributions, we have the following three cases:

Case 1: If both $\mathbf{X}$ and $\mathbf{X}^*$ are continuous.

(B2) reduces to the classical measurement error model by specifying $\mathbf{A}$ as the identity matrix and $\mathbf{B} \sim N(\mathbf{0}, \mathbf{\Sigma}_\epsilon)$ with the covariance matrix $\mathbf{\Sigma}_\epsilon$ and is independent of $\mathbf{X}$.

Case 2: If components in $\mathbf{X}$ and $\mathbf{X}^*$ are binary

Specifying $\mathbf{A} = \text{diag}(2S_1 - 1, \cdots, 2S_p - 1)$ and $\mathbf{B} = (1 - S_1, \cdots, 1 - S_p)^\top$ yields the difference-in-difference measurement error model (e.g., [29]), where $S_r \in \{0, 1\}$ with $S_r = 1$ reflecting $X_r = X_r^*$ and $S_r = 0$ reflecting $X_r \neq X_r^*$ (misclassification) for $r = 1, \cdots, p$.

Case 3: X_r and X_r^* are count data and follow the Poisson distribution for $r = 1, \cdots, p$

We can specify $\mathbf{A}$ as the identity matrix and $\mathbf{B} = \mathbf{Z} - \mathbf{W}$, where $\mathbf{Z}$ is a vector with components following the Poisson distribution with parameter λ and $\mathbf{W}$ is a vector with components following the Binomial distribution with X_i and probability of success π.

Noting that there are parameters $\mathbf{\Sigma}_\epsilon$, S_r, λ, and π in Cases 1-3, they can be estimated only when auxiliary information is available (e.g., [12, 13]). If the dataset has no auxiliary information, then one may adopt sensitivity analyses, whose strategy is to specify several values to the parameters and examine the impact of various degrees of measurement error effects. Alternatively, if one has background knowledge of the dataset or wishes to examine some specific values, then those parameters can be particularly specified by users or data analysts. In this study, to focus the discussion on the estimation method and introduction of the R package `GUEST`, we take those parameters as known values.

B.3 Linear Discriminant Analysis

For $i = 1, \cdots, n$, let Y_i denote the nominal random variable with C different labels for $C \geq 2$. In addition, let n_c denote the sample size in the class c, such that $\sum\limits_{c=1}^{C} n_c = n$. We wish to build up the classification model, which aims to classify subjects into their desired class Y_i by taking

the variable $\mathbf{X}_i$ as the covariates. In supervised learning, linear discriminant analysis (LDA) is one of useful methods to do classification. Specifically, the linear discriminant function is defined as

$$\delta_c(\mathbf{X}) \triangleq \log(\pi_c) - \frac{1}{2}\boldsymbol{\mu}_c^\top \boldsymbol{\Theta} \boldsymbol{\mu}_c + \mathbf{X}^\top \boldsymbol{\Theta} \boldsymbol{\mu}_c \tag{B3}$$

for $c = 1, \cdots, C$, where $\pi_c \triangleq P(Y_i = c)$, $\boldsymbol{\mu}_c$ is the expectation of $\mathbf{X}$ under the class c, and $\boldsymbol{\Theta} \triangleq \boldsymbol{\Sigma}^{-1}$. π_c and $\boldsymbol{\mu}_c$ can be empirically estimated by $\widehat{\pi}_c = \frac{n_c}{n}$ and $\widehat{\boldsymbol{\mu}}_c \triangleq n_c^{-1} \sum\limits_{i:Y_i=c} \mathbf{X}_i$. In conventional LDA, $\boldsymbol{\Theta}$ is estimated by $\widehat{\boldsymbol{\Theta}} \triangleq \widehat{\boldsymbol{\Sigma}}^{-1}$, where $\widehat{\boldsymbol{\Sigma}}$ is the empirical estimate of the covariance matrix. Consequently, (B3) is estimated by

$$\widehat{\delta}_c(\mathbf{X}) \triangleq \log(\widehat{\pi}_c) - \frac{1}{2}\widehat{\boldsymbol{\mu}}_c^\top \widehat{\boldsymbol{\Theta}} \widehat{\boldsymbol{\mu}}_c + \mathbf{X}^\top \widehat{\boldsymbol{\Theta}} \widehat{\boldsymbol{\mu}}_c, \tag{B4}$$

and thus, the predicted class is obtained by

$$c^* = \underset{c=1,\ldots,C}{\operatorname{argmax}} \, \widehat{\delta}_c(\widetilde{\mathbf{X}}),$$

where $\widetilde{\mathbf{X}}$ represents the covariates from a new subject.

However, (B4) is sometimes unrealistic because (i) measurement error and (ii) high-dimensionality in covariates. The former may affect the classification result and the latter makes $\boldsymbol{\Sigma}$ singular and non-invertiable, yielding that $\boldsymbol{\Theta}$ cannot be estimated.

C Methodology

C.1 The Estimation Procedure for The Network Structure

In this section, we introduce our strategy to deal with measurement error and high-dimensionality when estimating the network structure and the precision matrix $\boldsymbol{\Theta}$. The key steps are displayed in Figure C1. Specifically, we first reduce the original $p \times p$ precision matrix to the $q \times q$ submatrix $\boldsymbol{\Theta}_I$ with $q < n$, so that $\boldsymbol{\Theta}_I$ contains pairs of dependent variables and independent pairs of variables can be excluded. After that, we do variable selection and estimation to derive the estimator of $\boldsymbol{\Theta}_I$, denoted by $\widehat{\boldsymbol{\Theta}}_I$. Finally, we obtain the desired estimator of $\boldsymbol{\Theta}$. All steps take measurement error correction into account. To the end, we discuss the detailed procedure in the following subsections.

[Figure C1 is about here]

C.1.1 Measurement Error Correction

The first step is to eliminate the measurement error effect. We adopt the regression calibration method (e.g., [6]), whose key idea is to replace error-prone variable X_r^* by the conditional expectation $E(X_r|X_r^*)$ for $r = 1, \cdots, p$. Moreover, following an idea in [6] and [13], $E(X_r|X_r^*)$ can be approximated by the best linear unbiased prediction:

$$E(X_r|X_r^*) = E(X_r) + \frac{\text{cov}(X_r, X_r^*)}{\text{var}(X_r^*)}\{X_r^* - E(X_r^*)\}. \tag{C1}$$

In particular, under three different cases in Section B.2, (C1) has the following expressions:

Case 1: if X_r and X_r^* are continuous, then we have that

$$E(X_r|X_r^*) = \mu_{X_r} + \frac{\sigma_{X_r}^2}{\sigma_{X_r^*}^2}(X_i^* - \mu_{X_r^*}),$$

where $\mu_{X_r} = E(X_r)$, $\mu_{X_r^*} = E(X_r^*)$, $\sigma_{X_r}^2 = \text{var}(X_r)$, and $\sigma_{X_r^*}^2 = \text{var}(X_r^*)$.

Case 2: if X_r and X_r^* are binary, then we have that

$$E(X_r|X_r^*) = \frac{X_r^* + S_r - 1}{2S_r - 1}.$$

Case 3: if X_r and X_r^* are count, then we can obtain that

$$E(X_r|X_r^*) = \frac{\mu_{X_r^*} - \lambda}{1 - \pi} + \frac{\nu(\mu_{X_r^*} - \lambda)}{\lambda + \frac{3\pi+1}{1-\pi}(\mu_{X_r^*} - \lambda)}(X_r^* - \mu_{X_r^*})$$

with $\nu = \frac{\mu_{X_r^*} - \lambda}{1-\pi}$.

To the end, we use the new symbol $\widehat{X}_r$ to denote $E(X_r|X_r^*)$ with the parameters $\sigma_{X_r^*}^2$ and $\mu_{X_r^*}$ being empirically estimated by $\{X_{ir}^* : i = 1, \cdots, n\}$, and define a p-dimensional vector of the corrected variables $\widehat{\mathbf{X}} \triangleq (\widehat{X}_1, \cdots, \widehat{X}_p)^\top$. Moreover, define $\widehat{\mathbf{X}}_i \triangleq (\widehat{X}_{i1}, \cdots, \widehat{X}_{ip})^\top$ as the ith subject of $\widehat{\mathbf{X}}$ for $i = 1, \cdots, n$.

C.1.2 Feature Screening for Pairwise Dependence

Next, we adopt the feature screening technique to reduce the dimension of variables. Specifically, define a set

$$\mathcal{P} \triangleq \{(k, l) : X_k \text{ and } X_l \text{ are dependent for } k \neq l\}$$

that contains dependent pairs and its size is assumed to be smaller than the sample size due to sparsity. To estimate $\mathcal{P}$ and screen out independent pairs, we employ the Chatterjee's measure [7] with measurement error corrected:

$$\xi(\widehat{X}_k, \widehat{X}_l) = \frac{\int \text{var}[E\{I(\widehat{X}_l \geq t)|\widehat{X}_k\}]d\mu_l(t)}{\int \text{var}\{I(\widehat{X}_l \geq t)\}d\mu_l(t)}, \tag{C2}$$

where $\mu_l(\cdot)$ is the law of $\widehat{X}_l$. Based on (C2), we define $\omega_{kl} = \max\left\{\xi(\widehat{X}_k, \widehat{X}_l), \xi(\widehat{X}_l, \widehat{X}_k)\right\}$, and let $\widehat{\omega}_{kl}$ denote the resulting estimator that is determined by the rank-based approach (e.g., [7]). Consequently, $\mathcal{P}$ can be estimated by

$$\widehat{\mathcal{P}} = \{(k,l) : \widehat{\omega}_{kl} > \zeta \text{ for } k, l = 1, \cdots, p\}, \tag{C3}$$

where ζ is a user-specified thresholding positive constant. As commented in [9], $\widehat{\mathcal{P}}$ refers to the feature screening step, and we have only $\widehat{\mathcal{P}} \supseteq \mathcal{P}$ with probability approaching 1. It indicates that some independent pairs may be included due to weak signal $\widehat{\omega}_{kl}$.

C.1.3 Boosting Estimation

In this section, we follow the idea of the neighbourhood selection approach (e.g., [12, 33]) to estimate θ_{kl} and the netwrok strucutre. Let $\mathcal{V}_I$ denote the subset of $\mathcal{V}$ and it satisfies $\widehat{\mathcal{P}} \subset \mathcal{V}_I \times \mathcal{V}_I$. Define $\boldsymbol{\theta}_r \triangleq (\theta_{rl} : l \in \mathcal{V}_I \setminus \{r\})^\top$ as the $(|\mathcal{V}_I|-1)$-dimensional vector of parameters that is also the rth column in $\boldsymbol{\Theta}_I$. For a fixed $r \in \mathcal{V}_I$, the conditional probability of r given $\mathcal{V}_I \setminus \{r\}$ under (B1) is given by

$$\mathbb{P}_{\boldsymbol{\theta}_r}\left(X_r|\mathbf{X}_{\mathcal{V}_I\setminus\{r\}}\right) \propto \exp\left\{\sum_{l\in\mathcal{V}_I\setminus\{r\}} \theta_{rl}X_rX_l - \mathfrak{K}\left(\sum_{l\in\mathcal{V}_I\setminus\{r\}} \theta_{rl}X_l\right)\right\}, \tag{C4}$$

where $\mathbf{X}_{\mathcal{V}_I\setminus\{r\}} \triangleq (X_j : j \in \mathcal{V}_I \setminus \{r\})^\top$ and $\mathfrak{K}(\cdot)$ is the normalizing function that ensures (C4) to be integrated as 1. Due to the appearance of measurement error, we replace X_k by (C1), then under the sample with size n, the estimating function of (C4) is defined by the derivative of the log-likelihood function of (C4) with respect to $\boldsymbol{\theta}_r$, yielding that

$$\mathbf{S}(\boldsymbol{\theta}_r) = -\frac{1}{n}\sum_{i=1}^{n} \widehat{\mathbf{X}}_{i,\mathcal{V}_I\setminus\{r\}}\left\{\widehat{X}_{ir} - \mathfrak{K}'\left(\sum_{j\in V_I\setminus\{r\}} \theta_{rj}\widehat{X}_{ij}\right)\right\}, \tag{C5}$$

where $\widehat{\mathbf{X}}_{i,\mathcal{V}_I\setminus\{r\}} \triangleq (\widehat{X}_{ij} : j \in \mathcal{V}_I \setminus \{r\})^\top$.

Finally, to further retain dependent pairs and estimate the associate parameters from the estimating function (C5), we adopt the boosting algorithm, where the pseudo code is placed in Algorithm 1. Specifically, we start the algorithm by taking a zero vector as the initial value. For the $(t-t)$th iteration with the iterated value $\boldsymbol{\theta}_r^{(t-1)}$, let $\boldsymbol{\Delta}_r^{(t-1)} \triangleq \mathbf{S}(\boldsymbol{\theta}_r^{(t-1)})$ denote the $(|\mathcal{V}_I|-1)$-dimensional vector that is given by $\mathbf{S}(\boldsymbol{\theta}_r)$ evaluated at $\boldsymbol{\theta}_r^{(t-1)}$. After that, we determine the active set $\mathcal{J}_r^{(t-1)}$ that collects indices satisfying $\left|\widehat{\omega}_{rj}\Delta_{rj}^{(t-1)}\right| \geq \tau \max_{j'} \left|\widehat{\omega}_{rj'}\Delta_{rj'}^{(t-1)}\right|$, where $\widehat{\omega}_{kl}$ is defined in (C3) and is taken as the weight, $\Delta_{rj}^{(t-1)}$ is the jth component in $\boldsymbol{\Delta}_r^{(t-1)}$, and $\tau \in [0,1]$ is a pre-specified constant used to retain indices. As a result, $\mathcal{J}_r^{(t-1)}$ reflects the dependent vertexes with the vertex r. After that, we update θ_{rj} for all $j \in \mathcal{J}_r^{(t-1)}$ with the increment $\eta \cdot \text{sign}(\widehat{\omega}_{rj}\Delta_{rj}^{(t-1)})$, where η is the pre-specified small constant. After T iterations, we obtain the final estimator denoted by $\widehat{\boldsymbol{\theta}}_r$, which is rth column in $\boldsymbol{\Theta}_I$. Finally, running the same iterations for all $r \in \mathcal{V}_I$ yields the final estimator, which is denoted by $\widehat{\boldsymbol{\Theta}}_I$.

Algorithm 1: Boosting for detecting network structure

```
for r ∈ V_I do
    Set θ_r^(0) = 0_{|V_I|-1} ;
    for iteration t with t = 1, ··· , T do
        Step 1: compute Δ_r^(t-1) ≜ S(θ_r^(t-1)), where S(θ_r) is defined in (C5);
        Step 2: determine J_r^(t-1) = { j : |ω̂_rj Δ_rj^(t-1)| ≥ τ max_j' |ω̂_rj' Δ_rj'^(t-1)| };
        Step 3: update θ_rj^(t) = θ_rj^(t-1) + η · sign(ω̂_rj Δ_rj^(t-1)) for all j ∈ J_r^(t-1),
        and define θ_r^(t) = (θ_rj^(t) : j ∈ V_I\{r})^⊤;
    The estimator for a fixed row : θ̂_r ≜ θ_r^(T).
The final estimator : Θ̂ ≜ Diag(Θ̂_I, 0).
```

C.2 Classification with Error-Prone Variables

In the presence of measurement error and high-dimensionality, we have to adjust (B3) with those complex features taken into account. First, to deal with measurement error, we can replace $\mathbf{X}$ by $\widehat{\mathbf{X}}$. In addition, (C1) implies that $E(\mathbf{X}) = E(\widehat{\mathbf{X}})$, then $\boldsymbol{\mu}_c$ can be estimated by $\widehat{\boldsymbol{\mu}}_c^* \triangleq n_c^{-1} \sum_{i:Y_i=c} \widehat{\mathbf{X}}_i$. Second, the impact of high-dimensionality is on the estimation of $\boldsymbol{\Theta}$, which can be solved by the estimation procedure in Section C.1. Consequently, the modified linear discriminant function is given by

$$\widehat{\delta}_c^*(\widehat{\mathbf{X}}) \triangleq \log(\widehat{\pi}_c) - \frac{1}{2}\widehat{\boldsymbol{\mu}}_c^{*\top}\widehat{\boldsymbol{\Theta}}\widehat{\boldsymbol{\mu}}_c^* + \widehat{\mathbf{X}}^\top\widehat{\boldsymbol{\Theta}}\widehat{\boldsymbol{\mu}}_c^*, \tag{C6}$$

and thus, the predicted class for a new subject with the covariates $\widetilde{\mathbf{X}}$ is obtained by

$$c^* = \underset{c=1,\ldots,C}{\operatorname{argmax}} \widehat{\delta}_c(\widetilde{\mathbf{X}}).$$

D Illustration of The R Package GUEST

In this section, we introduce the R package `GUEST`. There are two main functions `boost.graph` and `LDA.boost` in this package, where the former is used to implement the estimation procedure in Section C.1 to derive the network structure, and the latter is used to construct the linear discriminant function in Section C.2 and make the classification. To the end, we describe the usage of two functions, their arguments, and outputs.

D.1 boost.graph

This function aims to implement the estimation procedure in Section C.1 to detect the network structure and estimate the precision matrix. To apply the Chatterjee's measure (C2), this function is only equipped with the R package `XICOR`. In addition, two R packages `network` and `GGally` are implemented to display the resulting network structure of random variables. The usage is given by

```
boost.graph(data, ite1, ite2, ite3, thre, select = 0.9, inc = 10^(-3),
 sigma_e = 0.6, q = 0.8, lambda = 1, pi = 0.5, rep = 100, cor = TRUE),
```

where the arguments include

- `data`: An $n \times p$ matrix of random variables, whose distributions can be continuous, discrete, or mixed. Here n and p represent the sample size and the dimension of variables, respectively.
- `ite1`: The number of iterations for continuous variables.
- `ite2`: The number of iterations for binary variables.
- `ite3`: The number of iterations for count variables.
- `thre`: The threshold value ζ for feature screening, whose value should be between 0 and 1.
- `select`: The threshold constant τ in the boosting algorithm, whose value should be between 0 and 1. The default value is 0.9.

- `inc`: The learning rate of the increment η in the boosting algorithm, which should be a small value. The default value is 0.001.
- `sigma_e`: The common value in the diagonal covariance matrix $\mathbf{\Sigma}_\epsilon$ in the classical measurement error model when `data` are continuous. The default value is 0.6.
- `q`: The common value used to characterize misclassification for binary random variables. The default value is 0.8.
- `lambda`: The parameter λ of the Poisson distribution, which is used to characterize error-prone count random variables. The default value is 1.
- `pi`: The probability π in the Binomial distribution, which is used to characterize error-prone count random variables. The default value is 0.5.
- `rep`: The number of bootstrapping iterations. The default value is 100.
- `cor`: Measurement error correction when estimating the precision matrix. The default value is TRUE.

The argument `data` contains p-dimensional random vector with sample size n. The variables can be error-prone or precisely measured, and the resulting correction can be specified by the argument `cor`. Three arguments `ite1`, `ite2`, and `ite3` refer to T in Algorithm 1 and are user-specified. They are used to run iterations for different distributions of random variables in `data`. The argument `thre` is the value ζ in $\widehat{\mathcal{P}}$ and is user-specified. Two arguments `select` and `inc` are τ and η in Algorithm 1, and are used to retain informative pairs and update the estimated values, respectively. Regarding parameters in measurement error models in Cases 1-3, users can specify values in `sigma_e`, `q`, `lambda`, and `pi`.

Based on the computation, we can obtain the following outputs:

- `w`: The estimator of the precision matrix.
- `p`: The chosen pairs obtained by the feature screening.
- `xi`: The weights sorted with pairs in `p`.
- `g`: The visualization of the estimated network structure determined by `w`.

Basically, this function provides pairs `p` in $\widehat{\mathcal{P}}$ and the corresponding values $\widehat{\omega}_{kl}$ that is also weights in Step 2 of Algorithm 1. In addition, the function gives the estimated precision matrix that is used to reflect the network structure of variables. The figure of network structure is also displayed for the visualization.

D.2 `LDA.boost`

```
LDA.boost(data, resp, theta, sigma_e = 0.6, q = 0.8, lambda = 1, pi = 0.5),
```

where the arguments `sigma_e`, `q`, `lambda`, and `pi` are the same as those in the function `boost.graph`. In addition, the remaining arguments are

- `data`: An $n \times p$ matrix of random variables, whose distributions can be continuous, discrete, or mixed.
- `resp`: An n-dimensional vector of categorical random variables, which is the response in the data.
- `theta`: The estimator of the precision matrix.

Three arguments are used to define the linear discriminant function (B4). The argument `theta` can be specified by users, but here we recommend to use the estimator derived by the function `boost.graph`. Based on the implementation, this function gives the following results:

- `score`: The value of the linear discriminant function (C6) with the estimator of the precision matrix accommodated.
- `class`: The result of predicted class for subjects.

E Analysis of Blue Cell Tumors Gene Expression Data

We apply the R package `GUEST` to analyze the small round blue cell tumors (SRBCT) gene expression data that were described in [24] and were also explored by some methods, such as [14], [23], and [30]. The full dataset is available at `https://CRAN.R-project.org/package=plsgenomics` with the data named SRBCT. The SRBCT dataset includes four similar childhood tumors: Ewing sarcoma (EWS), Burkitt lymphoma (BL), neuroblastoma (NB), and rhabdomyosarcoma (RMS), and they are labeled by 1, 2, 3, and 4, respectively. The sample size is $n = 83$, which is divided by $n_1 = 29$ in EWS, $n_2 = 11$ in BL, $n_3 = 18$ in NB, and $n_4 = 25$ in RMS. In addition, there are 2308 gene expression values, whose names can be found on the same website. Before analyzing the data, we first do standardization, such that each variable has empirical mean 0 and empirical variance 1. The following code shows the data setup:

```
library(plsgenomics)
data(SRBCT) # load data set
```

```
X = SRBCT$X
X_new = scale(X)
Y = SRBCT$Y
```

We have two targets in this data analysis: first, we aim to identify the network structure and estimate $\mathbf{\Theta}$ for gene expression variables; second, we wish to use gene expression values to classify subjects into their desired EWS, BL, NB, or RMS classes. As commented by [12], gene expressions are possibly contaminated with measurement error. Thus, we require to implement two functions in the R package `GUEST` to analyze this data.

To deal with the first target, we use the classical measurement error model with the covariance matrix $\mathbf{\Sigma}_\epsilon$ to characterize error-prone gene expressions because gene expressions are continuous variables. Since there is no additional information to $\mathbf{\Sigma}_\epsilon$, we primarily conduct sensitivity analyses by specifying $\mathbf{\Sigma}_\epsilon$ as a diagonal matrix with common entries $\sigma_\epsilon = 0.15, 0.35, 0.55, 0.75$ or 0.95. The parameters in Section C.1 are separately specified as $\zeta = 0.09$, $\tau = 0.9$ and $\eta = 0.001$. Detailed code demonstration is given below:

```
library(network)
library(GGally)
library(GUEST)
c1 = 0.15
## c1 can be replaced by 0.35, 0.55, 0.75, or 0.95 for the
# implementation of sensitivity analyses

result4_1 = boost.graph(data = X_new, ite1 = 1, ite2 = 0, thre = 0.09,
                        sigma_e = c1, rep = 1)
Strue4_1 = result4_1$w
temp4_1=Strue4_1[1:50,1:50]
net = temp4_1
net = network(net, directed = FALSE)
network.vertex.names(net)=paste0("X",network.vertex.names(net))
graph4_1 = ggnet2(net,size = 3, node.color = "lightgray", label = T,
                  label.size = 3, mode = "circle")
```

In addition to the implementation of the R package `GUEST`, we primarily examine the existing R packages `glasso`, `huge`, `space`, and `QUIC` listed in Table A1 to make the comparisons since some R packages, such as `clime`, require unexpectedly longer computation times and fails to estimate $\mathbf{\Theta}$ due to high-dimensionality. Moreover, to see the impact of the ignorance

of a sparse gene network, we also examine the regularized LDA (RLDA) method proposed by [27]. All existing methods do not take measurement error effects into account.

To demonstrate the estimated graphs clearly and to ease of discussion, we simply display the first 50 variables X_1-X_{50} in Figure E1. We can see that there are some edges that are detected by two packages `glasso` and `GUEST`, such as (X_{47}, X_{11}) and (X_{47}, X_{26}), where X_{47}, X_{11}, X_{26} are "heterogeneous nuclear ribonucleoprotein C", "large ribosomal protein P0", and "Human 90-kDa heat-shock protein gene", respectively; a pair (X_{49}, X_{25}) is only detected by the R packages `GUEST` and `space`, where X_{49} and X_{25} are "lactate dehydrogenase A" and "calmodulin 2", respectively. Moreover, it is also notable to see that some pairs are uniquely detected by the R package `GUEST`. For example, when $\sigma_\epsilon = 0.15, 0.35$, or 0.55, pairs (X_{49}, X_{26}), (X_{35}, X_7), (X_{35}, X_8), and (X_{35}, X_{11}) are identified, where X_{35}, X_7, and X_8 represent "adenine nucleotide translocator 3 (liver)", "guanine nucleotide binding protein (G protein)", and "pre-mRNA splicing factor SF3a (120 kDa subunit)", respectively. On the other hand, when $\sigma_\epsilon = 0.75$ or 0.95, a pair (X_{31}, X_{23}) is detected, where X_{31} and X_{23} are "glycogen synthase kinase 3 beta" and "glutamic-oxaloacetic transaminase 1", respectively. The detection of pairwise interactions seems to be affected by various magnitudes of measurement error effects.

[Figure E1 is about here]

The second target aims to handle multiple classification. We implement the linear discriminant function (C6) with the estimator of $\mathbf{\Theta}$ derived by the RLDA method and R packages `glasso`, `huge`, `space`, `QUIC`, and `GUEST` accommodated. For a subject i with $i = 1, \cdots, n$, we denote $\widehat{Y}_i \in \{1, 2, 3, 4\}$ as the predicted class label determined by the linear discriminant function, and denote $Y_i \in \{1, 2, 3, 4\}$ as the true class label with $Y_i = 1$ representing that the the patient i in in EWS, $Y_i = 2$ reflecting that the patient i belongs to BL, $Y_i = 3$ reflecting that the patient i is in NB, and $Y_i = 4$ reflecting that the patient i is in RMS. To assess the performance of the classification, we examine Precision (PRE), Recall (REC), and F-values (e.g., [10]). Specifically, for the kth class with $k = 1, 2, 3, 4$, the true positive (TP), the false positive (FP), and the false negative (FN) are respectively defined as

$$\mathrm{TP}_k = \sum_{i=1}^{n} I(Y_i = k, \widehat{Y}_i = k),\ \mathrm{FP}_k = \sum_{i=1}^{n} I(Y_i \neq k, \widehat{Y}_i = k),\ \text{and } \mathrm{FN}_k = \sum_{i=1}^{n} I(Y_i = k, \widehat{Y}_i \neq k).$$

After that, values of precision and recall for the kth class with $k = 1, 2, 3, 4$ are respectively given by

$$\mathrm{PRE}_k = \frac{\mathrm{TP}_k}{\mathrm{TP}_k + \mathrm{FP}_k} \text{ and } \mathrm{REC}_k = \frac{\mathrm{TP}_k}{\mathrm{TP}_k + \mathrm{FN}_k}, \tag{E1}$$

which yield the values of overall precision, recall:

$$\mathrm{PRE} = \frac{1}{4}\sum_{k=1}^{4}\mathrm{PRE}_k \text{ and } \mathrm{REC} = \frac{1}{4}\sum_{k=1}^{4}\mathrm{REC}_k. \tag{E2}$$

By (E2), the F-value is defined as

$$\mathrm{F} = 2\,\frac{\mathrm{PRE}\times\mathrm{REC}}{\mathrm{PRE}+\mathrm{REC}}. \tag{E3}$$

As commented in [10, 11], higher values of (E2) and (E3) reflect more accurate classification.

To implement the computation, we demonstrate the following code:

```
temp4_1 = Strue4_1
temp4_1 = temp4_1 + diag(1,2308,2308)
result_b1 = LDA.boost(X_new, Y, temp4_1)

TP1 = 0; TP2 = 0; TP3 = 0; TP4 = 0  # true positive for 4 classes
FN1 = 0; FN2 = 0; FN3 = 0; FN4 = 0  # false negative for 4 classes
FP1 = 0; FP2 = 0; FP3 = 0; FP4 = 0  # false positive for 4 classes

## Computing TP, FN, and FP
for (i in 1:length(Y)){
  TP1=TP1+sum(result_b1$class[i]==1 & Y[i]==1)
  FN1=FN1+sum(result_b1$class[i]!=1 & Y[i]==1)
  FP1=FP1+sum(result_b1$class[i]==1 & Y[i]!=1)

  TP2=TP2+sum(result_b1$class[i]==2 & Y[i]==2)
  FN2=FN2+sum(result_b1$class[i]!=2 & Y[i]==2)
  FP2=FP2+sum(result_b1$class[i]==2 & Y[i]!=2)

  TP3=TP3+sum(result_b1$class[i]==3 & Y[i]==3)
  FN3=FN3+sum(result_b1$class[i]!=3 & Y[i]==3)
  FP3=FP3+sum(result_b1$class[i]==3 & Y[i]!=3)

  TP4=TP4+sum(result_b1$class[i]==4 & Y[i]==4)
  FN4=FN4+sum(result_b1$class[i]!=4 & Y[i]==4)
```

```
  FP4=FP4+sum(result_b1$class[i]==4 & Y[i]!=4)
}

## Computing PRE and REC for 4 classes
P1_b1 = TP1/(TP1+FP1); R1_b1 = TP1/(TP1+FN1)
P2_b1 = TP2/(TP2+FP2); R2_b1 = TP2/(TP2+FN2)
P3_b1 = TP3/(TP3+FP3); R3_b1 = TP3/(TP3+FN3)
P4_b1 = TP4/(TP4+FP4); R4_b1 = TP4/(TP4+FN4)
```

Numerical results of (E1), (E2), and (E3) are summarized in Table E1. We find that the R package `GUEST` under various σ_ϵ outperforms the result derived by the existing methods or packages considered in Table A1. Specifically, it is clear to see that our method provides accurate classification with higher values of criteria (E1), (E2), and (E3) except for precision for NB and recall for RMS. Accompanied with the finding in network structure identification, interactions of "adenine nucleotide translocator 3 (liver)" with "guanine nucleotide binding protein (G protein)", "pre-mRNA splicing factor SF3a (120 kDa subunit)", and "large ribosomal protein P0" might be key ingredient to improve the classification result, which has not found in the existing literature (e.g., [14]; [23]; [30]). Moreover, based on sensitivity analyses, we find that the R package `GUEST` induces the most accurate classification result when $\sigma_\epsilon = 0.55$, which suggests that the magnitude of measurement error is about $\sigma_\epsilon = 0.55$, and implementing $0.15, 0.35$ or 0.75, 0.95 may cause "underestimation" or "overestimation" for σ_ϵ.

In contrast, the implementation of the existing methods and packages shows the impacts of measurement error and high-dimensionality. In particular, the RLDA method, which ignores the feature of sparse network structure, has the worst performance of the classification. It may show that gene expressions in the SRBCT data might have network structure, which affects the classification result. Moreover, unlike other existing methods [14], [23], and [30] that adopted dimension reduction or variable selection for identifying the main effects, our approach identifies informative pairs of gene expressions and then adopts the gene network to classify tumors.

[Table E1 is about here]

F Simulation Studies

In this section, we conduct simulation studies to further assess the performance of the proposed method and demonstrate the implementation of the R package `GUEST`.

F.1 Simulation Setup

Let $n = 500$ denote the sample size and let $p = 500, 1000$ be the dimension of a random vector. In our studies, we primarily examine two scenarios. The first scenario considers the *sparse* matrix $\mathbf{\Theta}$. Among p variables, the first 12 variables form four different network structures displayed in Figure F1, and the remaining $p - 12$ variables are independent. For each graph, we specify $\mathbf{X}$ to follow the normal, binomial, or Poisson distributions. Let $\mathbf{\Theta}_0 = [\theta_{0,kj}]$ denote the true value of the parameter that reflects network structures in Figure F1, i.e., $\theta_{0,kj} = 1$ (or 0) if X_j and X_k are (or are not) connected by an edge. Given required setups, the lattice, hub, and scale-free structures in Figure F1 can be generated by the R package `XMRF` [36], where the scale-free structures are differently generated at each generation by the R package `XMRF`. The generated synthetic data are regarded as the unobserved variable $\mathbf{X}$. In addition to $\mathbf{X}$, we also independently generate the binary random variable Y with the proportion 0.5.

[Figure F1 is about here]

The second scenario examines the *non-sparse* matrix $\mathbf{\Theta}$. Following the similar setting in [27], we particularly consider the continuous random vector $\mathbf{X}$ following the normal distribution with mean zero and the covariance matrix $\mathbf{\Sigma}_X$ specifying 1 and 0.8 in diagonal and non-diagonal entries, respectively. It gives that $\mathbf{\Theta}_0 \equiv \mathbf{\Sigma}_X^{-1}$ with common diagonal entries 4.512 and non-diagonal entries -0.488.

Next, provided the unobserved variable $\mathbf{X}$ generated by two scenarios, we generate the error-prone variable $\mathbf{X}^*$ by the measurement error model (B2). Specifically, when $\mathbf{X}$ is continuous, then we generate $\mathbf{X}^*$ by Case 1 with $\mathbf{\Sigma}_\epsilon = 0.1\mathbf{I}_p$ or $0.3\mathbf{I}_p$, where $\mathbf{I}_p$ is the $p \times p$ identity matrix; when elements in $\mathbf{X}$ are binary, then we generate $\mathbf{X}^*$ by Case 2 with S_i generated by the Bernoulli distribution with probability $\gamma = 0.9$ or 0.85; and finally, if elements in $\mathbf{X}$ are count, then we generate $\mathbf{X}^*$ by Case 3 with $\pi = 0.5$ and $\lambda = 0.5$ or 0.8.

The following programming code demonstrates the data generation:

```
####### Generation of the synthetic data
#Basic setup
library(XMRF)
n = 500
q1 = 12
q2 = 488
p = q1+q2

#Data generation
```

```
G = XMRF.Sim(n , q1, model = "GGM", graph.type = "lattice")
X=t(G$X)
Strue = diag(1,p,p)
Strue[1:q1,1:q1]= G$B +diag(max((eigen(Strue))$values+0.1),q1)
X1 = mvrnorm(n, rep(0,q2), diag(1,q2,q2))
X = cbind(X,X1)
Y = rbinom(n,1,0.5)

# Measurement Error
c = 0.1
W = X + mvrnorm(n, rep(0,p), diag(c,p,p))
```

As discussed in Section C, we have two targets: (i) detection of graphical structure and (ii) classification with estimated precision matrix accommodated. We will implement two functions `boost.graph` and `LDA.boost` to achieve these two goals. Numerical results and detailed discussions are deferred to Sections F.2 and F.3, respectively.

To see the advantage of the R package `GUEST`, we follow the similar discussion in Appendix E and compare with the existing R packages `glasso`, `clime`, `huge`, `space`, and `QUIC`. In addition, we also examine the measurement error correction method proposed by [35] (p.369), whose idea is to replace the empirical estimate of the covariance matrix of the error-prone variable $\mathbf{X}^*$ by

$$\widehat{\mathbf{\Gamma}} \triangleq \frac{1}{n}\mathbf{X}^*\mathbf{X}^{*\top} - \mathbf{\Sigma}_\epsilon,$$

and then implement it to the glasso method in the R package `glasso`. We call this approach "the Wainwright's method".

F.2 Simulation Results: Estimation of Graphical Structure

Let $\widehat{\mathbf{\Theta}} \triangleq [\widehat{\theta}_{kj}]$ denote the resulting estimator of $\mathbf{\Theta}$. To assess the performance of detecting the network structure, we compute specificity (SPE) and sensitivity (SEN), which are respectively given by

$$\text{SPE} = \frac{\#\left\{(k,j) : \theta_{0,kj} \neq 0 \text{ and } \widehat{\theta}_{kj} \neq 0\right\}}{\#\left\{(k,j) : \theta_{0,kj} \neq 0\right\}} \quad \text{and} \quad \text{SEN} = \frac{\#\left\{(k,j) : \theta_{0,kj} = 0 \text{ and } \widehat{\theta}_{kj} = 0\right\}}{\#\left\{(k,j) : \theta_{0,kj} = 0\right\}}. \tag{F1}$$

SPE and SEN are between 0 and 1, and larger values of SPE and SEN indicate more accurate detection of network structures. To assess the performance of estimation, we compute

Frobenius norm and Kullback-Leibler (KL) divergence:

$$\|\triangle_{\mathbf{\Theta}}\|_F = \sqrt{\sum_{k=1}^{p}\sum_{j=1}^{p}(\widehat{\theta}_{kj} - \theta_{0,kj})^2} \text{ and } \text{LOSS}_{\text{KL}} = \log(|\mathbf{\Theta}_0|/|\widehat{\mathbf{\Theta}}|) + \text{trace}(\widehat{\mathbf{\Theta}}\mathbf{\Theta}_0^{-1}) - p, \quad \text{(F2)}$$

where $\triangle_{\mathbf{\Theta}} \triangleq \widehat{\mathbf{\Theta}} - \mathbf{\Theta}_0$.

The implementation of the R package GUEST is summarized as follows, where the additional function spe_sen_bias_kl is used to conduct (F1) and (F2), whose detailed code is placed in the GitHub to save the space in the main text; the link is given by https://github.com/lchen723/GUEST.git. Moreover, the implementations of other existing R packages are also placed in the GitHub.

```
########## GUEST Implementation
library(GUEST)
## Estimate $\Theta$
result1 = boost.graph(data = W, thre = 0.2, ite1 = 10, rep = 1,
           sigma_e = c, cor = T)
Theta = result1$w
## Compute evaluation criteria
spe_sen_bias_kl(Theta = Theta, Strue = Strue, p = 500)
## Draw an estimated graph
net_GUEST = network::network(Theta[1:12,1:12], directed = FALSE)
network::network.vertex.names(net_GUEST) =
   paste0("X",network::network.vertex.names(net_GUEST))
graph = GGally::ggnet2(net_GUEST, size=3, node.color = "lightgray",
        label=T, label.size = 3, mode = "circle")
proc.time()
```

We first display the estimated network structures of the first 12 variables X_1-X_{12} in Figure F2. When there is no network structure, i.e., Independence in Figure F1, most methods correctly detect unconnected pairs. In contrast, when network structures contain edges, i.e., Lattice and Hub in Figure F1, the R package GUEST successfully detects all informative edges; however, the other existing packages fail to identify the network structure. In particular, the R package glasso falsely excludes informative pairs while the R packages clime and space falsely include non-informative pairs. While the Wainwright's method and the R package huge have accurate detections for the Hub structure, some edges are still missing when estimating

the Lattice structure. It is also interesting to see that the R package `QUIC` is possible to detect no edges.

[Figure F2 is about here]

Tables F1-F4 summarize numerical results (F1) and (F2) for the settings in Section F.1, including sparse and non-sparse matrix $\boldsymbol{\Theta}$. We find that most methods produce estimation results, except for the R package `clime` and the Wainwright' method when $p = 1000$ for some settings. From the performance of the estimators, we observe that, except for the R packages `huge` and `space`, most methods have comparable $\|\triangle_{\boldsymbol{\Theta}}\|_F$ when $\mathbf{X}$ follows a normal distribution with network structures in $\boldsymbol{\Theta}$, which is due to that most R packages are designed to deal with the GGM. However, when $\boldsymbol{\Theta}$ is non-sparse, even though $\mathbf{X}$ follows the normal distribution, we find that values of $\|\triangle_{\boldsymbol{\Theta}}\|_F$ and LOSS_{KL} obtained by existing methods are extremely greater than those derived by the R package `GUEST`, which indicates that our package is also valid to handle te non-sparse matrix $\boldsymbol{\Theta}$. On the other hand, when $\mathbf{X}$ is either binary or count, we find that the Wainwright's method and most R packages induce tremendous biases because of larger values of $\|\triangle_{\boldsymbol{\Theta}}\|_F$ and LOSS_{KL}, which is not only due to impacts of measurement error effects, but also caused by non-normal distributions. In addition, the R package `GUEST` produces the smallest value of LOSS_{KL} regardless of distributions of $\mathbf{X}$ as well as the magnitudes of measurement error effects.

[Tables F1, F2, F3, and F4 are about here]

In addition, when the network structure is sparse, SPE and SEN produced by the R package `GUEST` are close or equal to 1 regardless of the distributions of $\mathbf{X}$, which reveal that `GUEST` detects informative/non-informative pairs of variables accurately. In summary, simulation results reveal that the R package `GUEST` is valid to detect the network structure and estimate $\boldsymbol{\Theta}$ regardless of types of network structures.

Finally, Tables F1-F4 also record the computation time obtained by the function `proc.time()`. Intuitively, the R package `GUEST` requires slightly longer but reasonable computational times because it implements the feature screening procedure and the boosting iterations in Algorithm 1. Specifically, as commented in Azadkia and Chatterjee (2021), (C2) has the computational complexity $O(n \log n)$, and $\widehat{\omega}_{kl}$ in (C3) should be computed for $1 \leq k < l \leq p$, reflecting that the dimension p and the sample size n may affect the computational time. In addition, $\widehat{\boldsymbol{\Theta}}_I$ is obtained by solving (C5) for all $r \in \mathcal{V}_I$ and T times iterations in Algorithm 1, which may affect the computational time. According to our additional survey by examining $n = 50$ and $p = 200$ with $p > n$ in the GitHub, the computational time can be faster, which suggests that the R package `GUEST` is still able to handle a dataset with moderately sized dimension p and obtain the estimation result under the reasonable computational time.

In contrast, the R packages `glasso`, `huge`, and `QUIC` directly estimate the matrix $\boldsymbol{\Theta}$, so that they are fast to obtain the estimation results, but they induce the biases easily because they primarily restrict in normally distributed data. In addition, it is also surprising to see that the R packages `clime` and `space` require the longer computational time to obtain the result, which might be caused by high-dimensional variables. In particular, the Wainwright's correction method and the R package `clime` are possible to produce no result when p is large enough.

F.3 Simulation Results: Classification

As mentioned in Section F.1, the second target is to do classification. We employ (C6) with $\widehat{\boldsymbol{\Theta}}$ being derived by the R packages and estimation methods in Section F.2. The following programming code shows the implementation of the R package `GUEST`; the other existing R packages are placed in the GitHub.

```
## GUEST Implementation
class_GUEST = LDA.boost(W, Y, Theta, sigma_e = 0.1)

## Computation of (12) and (13) with K=2;
TP = 0; FP = 0; FN = 0
for (i in 1:length(Y)){
  TP = TP+sum(class_GUEST$class[i]==0 & Y[i]==0)
  FN = FN+sum(class_GUEST$class[i]==1 & Y[i]==0)
  FP = FP+sum(class_GUEST$class[i]==0 & Y[i]==1)
    }
PRE = TP/(TP+FP); REC = TP/(TP+FN); F = 2*((PRE*REC)/(PRE+REC))
```

To assess the performance of the classification, we compute (E2) and (E3) with $K = 2$, and summarize numerical results in Tables F5-F8. In general, we observe that the R package `GUEST` performs the best with the largest F-value among all methods regardless of distributions of $\mathbf{X}$ and the sparsity of $\boldsymbol{\Theta}$ in the first and second scenarios in Section F.1. It shows that the R package `GUEST` is robust to yield accurate classification under various settings. In contrast, the classification results derived by existing R packages are comparable and are slightly worse than those determined by the R package `GUEST`.

[Tables F5, F6, F7, and F8 are about here]

G Summary and Extensions

In this paper, we introduce a new R package GUEST, whose main goal is to estimate network structures of ultrahigh-dimensional and error-prone variables. Unlike other existing R packages that primarily focus on continuous random variables, our package can handle various types of random variables, including binary or count data. In addition, our package can correct for measurement error effects. For the implementation of classification, our package provides the linear discriminant function with measurement error in variables corrected. With the estimated precision matrix accommodated, the classification result can be improved and the performance of our package is better than existing methods.

To address ultrahigh-dimensionality, the R package GUEST adopts the Chatterjee's measure (C2) to retain strongly dependent pairs of variables. As reminded by one referee, [1] proposed a measure to examine the conditional dependence, which is based on (C2) and is formulated by

$$\frac{\int E(\mathrm{var}[E\{I(Y \geq t)|X, Z\}|X])d\mu(t)}{\int E[\mathrm{var}\{I(Y \geq t)|X\}]d\mu(t)}, \tag{F3}$$

where Y, Z, and X are three random variables, and $\mu(\cdot)$ is the law of Y. (F3) primarily examines the conditional dependence of Y and Z, given X. Moreover, [1] also proposed the feature ordering by conditional independence (FOCI) method, which implements (F3) to select informative variables in regression models. Intuitively, one may expect to extend (F3) to detect network structures, but some modifications are required. Specifically, following the spirit of the conditional probability (C4), we can treat X_r as the response and take $\mathbf{X}_{\mathcal{V}\setminus\{r\}} \triangleq (X_1, \cdots, X_{r-1}, X_{r+1}, \cdots, X_p)^\top$ as the covariates for $r \in \mathcal{V}$, then one may adopt the FOCI method to detect X_l that is connected with X_r for $l \in \mathcal{V} \setminus \{r\}$. However, the corresponding parameters θ_{rl} in (C5) should be estimated by modifying ω_{kl} in Algorithm 1 with (C2) replaced by (F3). In addition, while the FOCI method ensures that the set of selected variables is sufficient, the resulting theoretical property requires some conditions that may not fit the current setting. For example, the theory of the FOCI method requires that the dimension p is quite large compared to the sample size n, i.e., $n \gg \log p$, but the setting in the current development is $p \gg n$. Hence, whether (F3) and the FOCI method are valid to handle the $p \gg n$ setting in the current manuscript is deserved to explore in the near future.

The current development of this package lies on p-dimensional random vector with measurement error. As discussed in [8], there are a lot of additional topics that can be explored based on the current ultrahigh-dimensional and error-prone settings. For example, multiple graphical models are challenging structures since they reflect heterogeneous data, i.e., the

same variables in several different categories. It would be interesting to extend the current estimation procedure in the R package GUEST to handle this complex structure with measurement error effects accommodated, and the resulting estimator can be expected to improve the performance of the classification in quadratic discriminant analysis.

Declarations

- Ethics approval and consent to participate: Not applicable
- Consent for publication: Not applicable
- Competing interests: The authors declare that they have no competing interests
- Funding: National Science and Technology Council of Taiwan (L.-P. Chen).
- Authors' contributions:
  - L.-P. Chen: paper preparation, writing, idea motivation, supervision, revision.
  - H.-S. Tsao: paper preparation, coding, idea motivation, revision.
- Availability of data and materials: The full dataset is available on the R CRAN `https://CRAN.R-project.org/package=plsgenomics`.
- Availability and requirements:
  - Project name: GUEST
  - Project home page: `https://cran.r-project.org/web/packages/GUEST/index.html`
  - Programming language: R
  - Other requirements: R 3.5.0 or higher
  - Operating system(s): Platform independent
  - License: GPL-2
  - Any restrictions to use by non-academics: No
- Acknowledgements: Not Applicable

References

[1] Azadkia, M. and Chatterjee, S. (2021). A simple measure of conditional dependence. *The Annals of Statistics*, 49, 3070-3102.

[2] Banerjee, O., Ghaoui, L. E., and d'Aspremont, A. (2008). Model selection through sparse maximum likelihood estimation for multivariate Gaussian or binary data. *Journal of Machine Learning Research*, 9, 485–516.

[3] Cai, T. Liu, W., and Luo, X. (2011). A constrained ℓ_1 minimization approach to sparse precision matrix estimation. *Journal of the American Statistical Association*, 106, 594–607.

[4] Cai, X., Bazerque, J. A., and Giannakis, G. B. (2013). Inference of gene regulatory networks with sparse structural equation models exploiting genetic perturbations. *PLOS Computational Biology*, 9, e1003068.

[5] Cai, T. Liu, W., and Luo, X. (2011). Package clime: constrained L1-minimization for inverse (covariance) matrix estimation. `https://CRAN.R-project.org/package=clime`.

[6] Carroll, R. J., Ruppert, D., Stefanski, L. A., and Crainiceanu, C. M. (2006). *Measurement Error in Nonlinear Model*. Chapman and Hall, Boca Raton, FL.

[7] Chatterjee, S. (2021). A new coefficient of correlation. *Journal of the American Statistical Association*, 116, 2009-2022.

[8] Chen, L.-P. (2024). Estimation of graphical models: An overview of selected topics. *International Statistical Review*, 92, 194-245.

[9] Chen, L.-P. (2023). A note of feature screening via a rank-based coefficient of correlation. *Biometrical Journal*, 65, 2100373.

[10] Chen, L.-P. (2022a). Network-based discriminant analysis for multiclassification. *Journal of Classification*, 39, 410-431.

[11] Chen, L.-P. (2022b). NetDA: An R package for network-based discriminant analysis subject to multi-label classes. *Journal of Probability and Statistics*, Article ID 1041752, 1-14.

[12] Chen, L.-P. and Yi, G. Y. (2022). De-noising analysis of noisy data under mixed graphical models. *Electronic Journal of Statistics*, 16, 3861-3909.

[13] Chen, L.-P. and Yi, G. Y. (2021). Semiparametric methods for left-truncated and right-censored survival data with covariate measurement error. *Annals of the Institute of Statistical Mathematics*, 73, 481–517.

[14] Chen, Q.-R., Vansant, G., Oades, K., Pickering, M., Wei, J. S., Song, Y. K., Monforte, J., and Khan, J. (2007). Diagnosis of the small round blue cell tumors using multiplex polymerase chain reaction. *Journal of Molecular Diagnostics*, 9, 80-88.

[15] Clemmensen, L. and Kuhn, M. (2016). Package sparseLDA: sparse discriminant analysis. `https://cran.r-project.org/package=sparseLDA`.

[16] Friedman, J., Hastie, T., and Tibshirani, R. (2008). Sparse inverse covariance estimation with the graphical lasso. *Biostatistics*, 9, 432–441.

[17] Friedman, J., Hastie, T., and Tibshirani, R. (2019). Package glasso: Graphical Lasso: Estimation of Gaussian Graphical Models. `https://CRAN.R-project.org/package=glasso`.

[18] Haslbeck, J. (2023). Package mgm: Estimating time-varying k-order mixed graphical models. `https://cran.r-project.org/package=mgm`.

[19] Højsgaard, S. (2023). Package gRim: Graphical interaction models. `https://cran.r-project.org/package=gRim`.

[20] Hsieh, C.-J., Sustik, M. A., Dhillon, I. S., and Ravikumar, P. (2014). QUIC: Quadratic approximation for sparse inverse covariance estimation. *Journal of Machine Learning Research*, 15, 2911-2947.

[21] James, G. M., Sabatti, C., Zhou, N., and Zhu, J. (2010). Sparse regulatory networks. *Annals of Applied Statistics*, 4, 663-686.

[22] Jiang, H., Fei, X., Liu, H., Roeder, K., Lafferty, J., Wasserman, L., Li, X., and Zhao, T. (2021). Package huge: High-dimensional undirected graph estimation. `https://cran.r-project.org/package=huge`.

[23] Karimi, S. and Farrokhnia, M. (2014). Leukemia and small round blue-cell tumor cancer detection using microarray gene expression data set. *Chemometrics and Intelligent Laboratory Systems*, 139, 6–14.

[24] Khan, J., Wei, J. S., Ringner, M., Saal, L. H., Ladanyi, M., Westermann, F., Berthold, F., Schwab, M., Antonescu, C. R., Peterson, C., and Meltzer, P. S. (2001). Classification

and diagnostic prediction of cancers using gene expression profiling and artificial neural networks. *Nature Medicine*, 7, 673-679.

[25] Khare, K., Oh, S.-Y., and Rajaratnam, B. (2015). A convex pseudolikelihood framework for high dimensional partial correlation estimation with convergence guarantees. *Journal of Royal Statistical Society, Series B*, 77, 803-825.

[26] Leclerc, R. D. (2008). Survival of the sparsest: robust gene networks are parsimonious. *Molecular Systems Biology*, 4 (1): 213.

[27] Mahadi, M., Ballal, T., Moinuddin, M., Al-Naffouri, T. Y., and Al-Saggaf, U. M. (2024). Regularized linear discriminant analysis using a nonlinear covariance matrix estimator. *IEEE Transactions on Signal Processing*, 72, 1049-1064.

[28] Mazumder, R. and Hastie, T. (2012). The graphical lasso: New insights and alternatives. *Electronic Journal of Statistics*, 6, 2125-2149.

[29] Negi, A. and Negi, D. S. (2022). Difference-in-differences with a misclassified treatment. arXiv:2208.02412

[30] Pal, N. R., Aguan, K., Sharma, A., Amari, S. (2007). Discovering biomarkers from gene expression data for predicting cancer subgroups using neural networks and relational fuzzy clustering. *BMC Bioinformatics*, 8:5.

[31] Peng, J., Wang, P., Zhou, N., and Zhu, J. (2009). Partial correlation estimation by joint sparse regression models. *Journal of the American Statistical Association*, 104, 735-746.

[32] Qiu, H., Han, F., Liu, H., and Caffo, B. (2016) Joint estimation of multiple graphical models from high dimensional time series. *Journal of the Royal Statistical Society Series B: Statistical Methodology*, 78, 487-504.

[33] Ravikumar, P., Wainwright, M. J., and Lafferty, J. (2010). High dimensional Ising model selection using ℓ_1-regularized logistic regression. *The Annals of Statistics*, 38, 1287-1319.

[34] Tsao, H.-S. and Chen, L.-P. (2024). Package GUEST: Graphical models in ultrahigh-dimensional and error-prone data via boosting algorithm. `https://cran.r-project.org/package=GUEST`.

[35] Wainwright, M. J. (2019). *High-Dimensional Statistics: A Non-Asymptotic Viewpoint.* Cambridge University Press, Cambridge.

[36] Wan, Y.-W., Allen, G. I., Baker, Y., Yang, E., Ravikumar, P., Anderson, M., and Liu, Z. (2016). XMRF: an R package to fit Markov Networks to high-throughput genetics data. *BMC Systems Biology*, 10(Suppl 3):69.

[37] Wang, L., Chen, Z., Wang, C. D., and Li, R. (2020). Ultrahigh dimensional precision matrix estimation via refitted cross validation. *Journal of Econometrics*, 215, 118-130.

[38] Witten, D. M. and Tibshirani, R. (2011). Penalized classification using Fisher's linear discriminant. *Journal of the Royal Statistical Society. Series B (Statistical Methodology)*, 73, 753-772.

[39] Yang, Y., Dai, H., and Pan, J. (2023). Block-diagonal precision matrix regularization for ultra-high dimensional data. *Computational Statistics and Data Analysis*, 179, 107630.

[40] Yuan, M. and Lin, Y. (2007). Model selection and estimation in the Gaussian graphical model. *Biometrika*, 94, 19-35 .

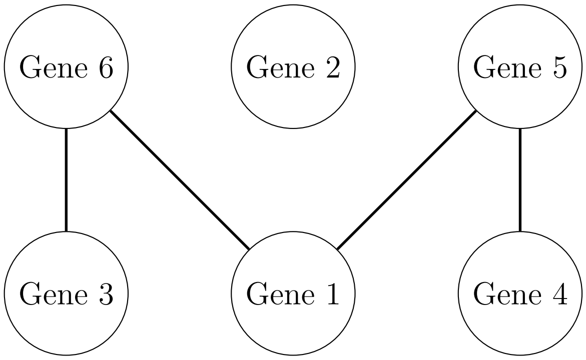

$$\boldsymbol{\Theta} = \begin{pmatrix} 0 & 0 & 0 & 0 & 1 & 1 \\ 0 & 0 & 0 & 0 & 0 & 0 \\ 0 & 0 & 0 & 0 & 0 & 1 \\ 0 & 0 & 0 & 0 & 1 & 0 \\ 1 & 0 & 0 & 1 & 0 & 0 \\ 1 & 0 & 1 & 0 & 0 & 0 \end{pmatrix}$$

A **B**

Figure B1: Illustrative diagrams of the network structure in gene expressions with $p = 6$. The panel A is the network structure of six gene expressions. The panel B is the adjacency matrix that reflects the network structure in the panel A.

$$\boldsymbol{\Theta} \xrightarrow{\text{ME + FS}} \left(\begin{array}{c|c} \boldsymbol{\Theta}_I & \mathbf{O}_{q\times(p-q)} \\ \hline \mathbf{O}_{(p-q)\times q} & \mathbf{O}_{(p-q)\times(p-q)} \end{array}\right) \xrightarrow{\text{ME + VS}} \left(\begin{array}{c|c} \widehat{\boldsymbol{\Theta}}_I & \mathbf{O}_{q\times(p-q)} \\ \hline \mathbf{O}_{(p-q)\times q} & \mathbf{O}_{(p-q)\times(p-q)} \end{array}\right) \triangleq \widehat{\boldsymbol{\Theta}}$$

Figure C1: Illustrative diagrams of the estimation steps for $\boldsymbol{\Theta}$. $\mathbf{O}_{q\times q}$ is the $q \times q$ zero matrix. ME is the measurement error correction, FS represents feature screening, and VS denotes variable selection.

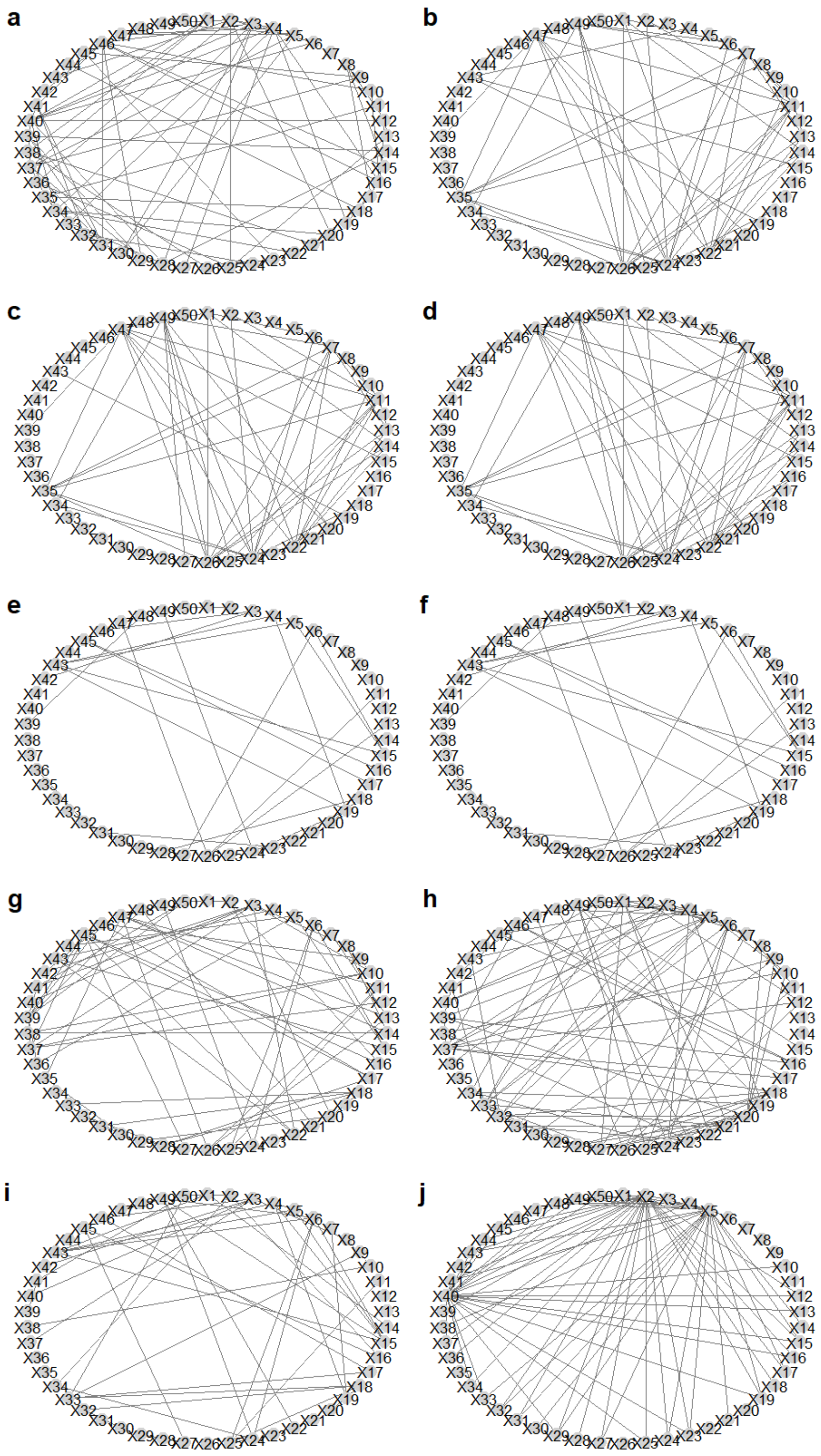

Figure E1: The visualization of estimated network structures of the SRBCT data in Section E. Graph a is derived by `glasso`; Graphs b-f are derived by `GUEST` with σ_ϵ being 0.15, 0.35, 0.55, 0.75, and 0.95 respectively. Graphs g, h, and i are determined by the R packages `huge`, `space`, and `QUIC`, respectively. Graph j is derived by RLDA.

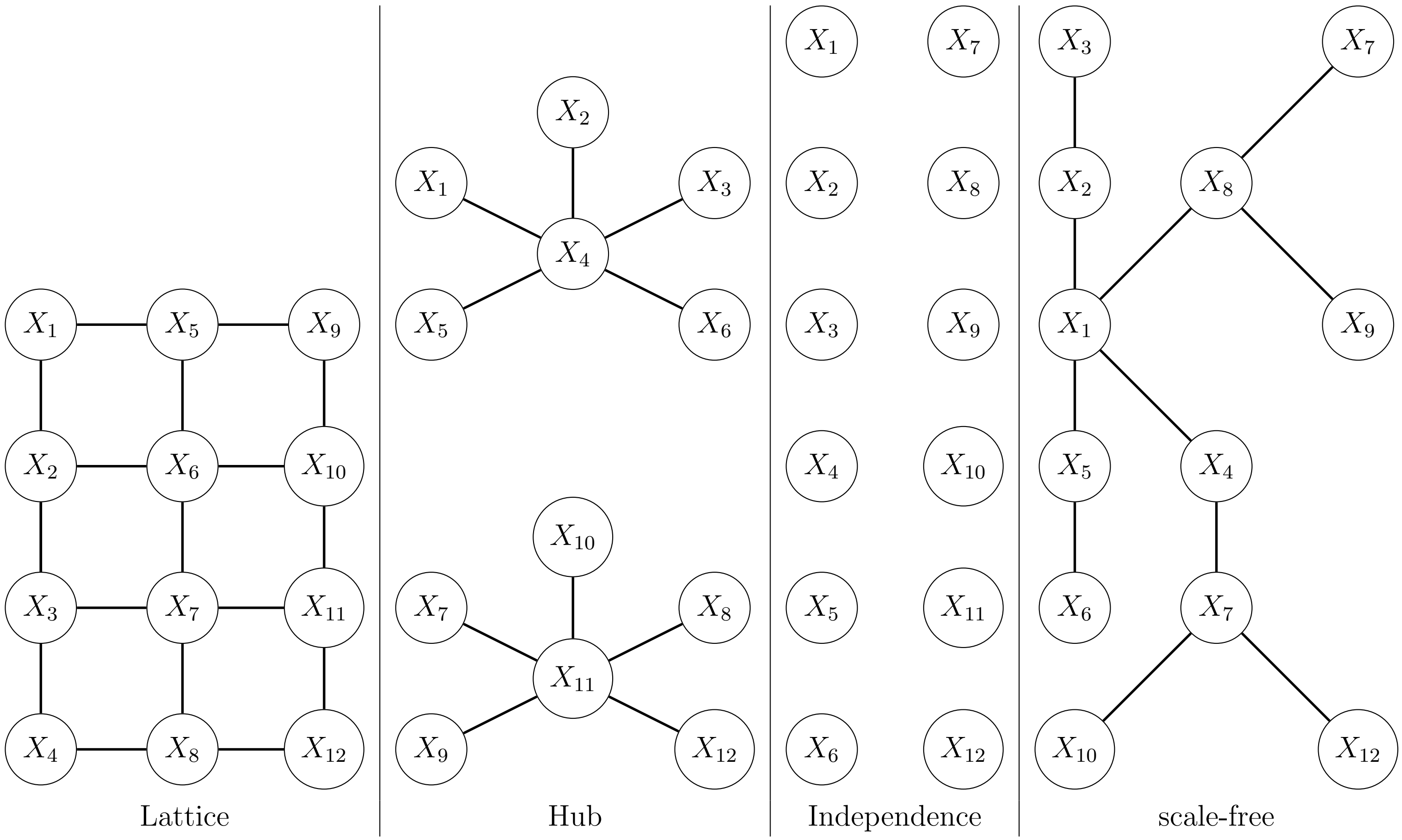

Figure F1: Various network structures in simulation studies. Here the "scale-free" network structure is the illustration; the network structure may be different from each data generation.

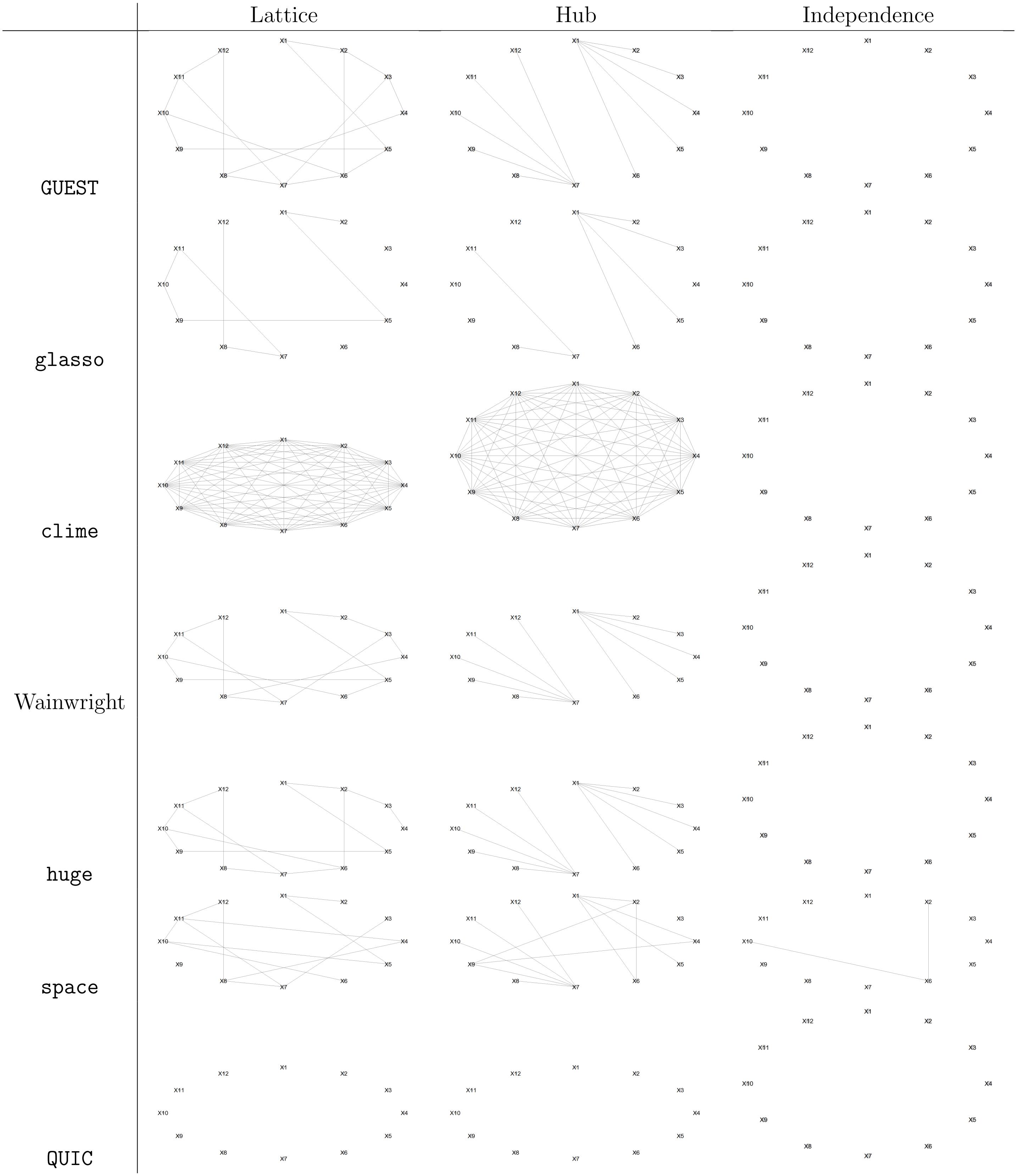

Figure F2: The visualization of estimated network structures of the simulated data in Section F.2

Table A1: Comparisons among existing packages. ME represents the measurement error correction, DIST represents the allowance of various distributions in random variables, HD is high-dimensionality, especially for the dimension is greater than the sample size ($p \gg n$), NL indicates nonlinear or nonparametric in variables, and Archive reflects whether the R package is archive.

Purpose	Package	ME	DIST	HD	NL	Archive
Graphical estimation	`glasso` [17]	×	×	×	×	×
	`clime` [5]	×	×	×	×	×
	`QUIC` [20]	×	×	×	×	✓
	`dpglasso` [28]	×	×	×	×	✓
	`XMRF` [36]	×	✓	×	×	✓
	`space` [31]	×	×	×	×	✓
	`gconcord` [25]	×	×	×	×	✓
	`gRim` [19]	×	✓	×	×	×
	`mgm` [18]	×	✓	×	×	×
	`huge` [22]	×	×	✓	✓	×
Classification	`sparseLDA` [15]	×	×	×	×	×
	`penalizedLDA` [38]	×	×	×	×	✓
Graphical estimation + Classification	`GUEST` [34]	✓	✓	✓	✓	×
	`NetDA` [11]	×	×	×	×	×

Table E1: Classification results for small round blue cell tumors gene expression data.

Criteria	`glasso`	`GUEST`					`huge`	`space`	`QUIC`	RLDA
		$\sigma_\epsilon = 0.15$	0.35	0.55	0.75	0.95				
PRE_1	0.963	1.000	1.000	1.000	1.000	1.000	1.000	0.000	1.000	0.000
PRE_3	1.000	1.000	1.000	1.000	1.000	1.000	0.917	0.121	0.917	0.059
PRE_3	0.947	0.857	0.857	0.947	0.900	0.900	0.900	0.000	0.947	0.417
PRE_4	0.923	1.000	1.000	1.000	1.000	1.000	1.000	0.000	1.000	0.000
PRE	0.958	0.964	0.964	0.987	0.966	0.966	0.931	0.000	0.966	0.000
REC_1	0.897	0.966	0.966	0.966	1.000	1.000	1.000	0.364	1.000	0.273
REC_2	1.000	1.000	1.000	1.000	1.000	1.000	1.000	0.000	1.000	0.556
REC_3	1.000	1.000	1.000	1.000	0.960	0.960	0.960	0.000	0.960	0.000
REC_4	0.960	0.920	0.920	1.000	0.975	0.975	0.954	0.030	0.966	0.119
REC	0.964	0.972	0.972	0.992	0.981	0.981	0.973	0.091	0.981	0.207
F	0.961	0.968	0.968	0.989	0.978	0.978	0.963	0.045	0.974	0.151

Table F1: Simulation results for the first scenario in Section F.1: graphical structure in Figure F1 under continuous random variables

Graph	p	Methods	$\boldsymbol{\Sigma}_\epsilon = 0.1\mathbf{I}_p$					$\boldsymbol{\Sigma}_\epsilon = 0.3\mathbf{I}_p$				
			SPE	SEN	$\Vert\triangle_{\boldsymbol{\Theta}}\Vert_F$	LOSS_{KL}	Time	SPE	SEN	$\Vert\triangle_{\boldsymbol{\Theta}}\Vert_F$	LOSS_{KL}	Time
Lattice	500	`glasso`	1.000	1.000	4.586	6.992	1.490	1.000	1.000	5.493	59.941	1.590
		`clime`	1.000	1.000	5.383	40.714	1487.580	1.000	1.000	5.291	41.622	1473.290
		`GUEST`	1.000	1.000	3.162	2.439	497.990	1.000	1.000	3.124	3.011	537.740
		Wainwright	0.993	1.000	4.935	42.955	1.650	1.000	1.000	6.758	111.717	1.640
		`huge`	1.000	1.000	23.501	499.388	1.840	1.000	1.000	23.446	499.069	1.590
		`space`	0.993	0.917	19.338	7.978	593.570	0.996	0.912	19.805	8.017	324.660
		`QUIC`	1.000	1.000	5.910	81.504	0.670	1.000	1.000	7.859	136.612	0.680
	1000	`glasso`	1.000	1.000	5.264	60.184	19.270	1.000	1.000	5.942	32.989	21.690
		`clime`	–	–	–	–	–	–	–	–	–	–
		`GUEST`	1.000	1.000	3.816	3.636	754.170	1.000	1.000	3.875	4.015	1246.420
		Wainwright	–	–	–	–	–	–	–	–	–	–
		`huge`	1.000	1.000	32.433	999.348	3.400	1.000	1.000	32.393	999.006	3.560
		`space`	0.973	0.999	6.949	5.555	2855.660	0.977	0.998	7.618	5.766	1203.750
		`QUIC`	1.000	1.000	6.727	152.750	5.090	1.000	1.000	9.498	255.942	5.660
Hub	500	`glasso`	1.000	1.000	3.346	14.839	1.720	1.000	1.000	4.603	67.547	1.940
		`clime`	1.000	1.000	4.309	45.620	1479.470	1.000	1.000	4.095	40.460	1487.580
		`GUEST`	1.000	1.000	2.585	2.967	233.310	1.000	1.000	2.697	3.019	429.080
		Wainwright	1.000	1.000	3.577	43.063	1.420	1.000	1.000	6.021	111.674	1.270
		`huge`	1.000	1.000	23.141	499.723	1.340	1.000	1.000	23.085	499.338	1.190
		`space`	0.996	0.912	19.394	7.903	262.660	1.000	0.924	17.866	7.605	362.240
		`QUIC`	1.000	1.000	4.842	81.439	0.370	1.000	1.000	7.059	136.523	0.580
	1000	`glasso`	1.000	1.000	4.064	60.483	19.270	1.000	1.000	3.917	31.991	21.720
		`clime`	–	–	–	–	–	–	–	–	–	–
		`GUEST`	1.000	1.000	4.030	3.738	962.000	1.000	1.000	3.470	3.660	1125.410
		Wainwright	–	–	–	–	–	–	–	–	–	–
		`huge`	1.000	1.000	33.498	1007.321	3.500	1.000	1.000	32.142	999.374	3.080
		`space`	0.998	0.967	14.239	6.814	752.750	0.998	0.913	19.873	7.383	1197.890
		`QUIC`	1.000	1.000	5.792	152.629	3.010	1.000	1.000	8.825	255.776	3.970

Independence	500	`glasso`	1.000	1.000	1.549	12.902	1.570	1.000	1.000	5.156	65.535	1.090
		`clime`	1.000	0.999	3.482	44.827	1496.500	1.000	1.000	5.590	36.756	831.880
		`GUEST`	1.000	1.000	1.172	0.345	110.460	1.000	1.000	4.928	0.818	480.170
		Wainwright	1.000	1.000	2.147	45.780	0.850	1.000	1.000	5.212	114.606	1.240
		`huge`	1.000	1.000	22.368	198.190	2.610	1.000	1.000	22.368	497.850	2.640
		`space`	1.000	0.990	8.365	1.289	187.120	1.000	0.989	9.026	1.270	196.070
		`QUIC`	1.000	1.000	3.568	79.053	0.200	1.000	1.000	6.034	134.397	0.470
	1000	`glasso`	1.000	1.000	2.854	63.590	19.290	1.000	1.000	3.869	28.537	21.500
		`clime`	–	–	–	–	–	–	–	–	–	–
		`GUEST`	1.000	1.000	1.916	0.273	1114.000	1.000	1.000	3.207	0.775	708.160
		Wainwright	–	–	–	–	–	–	–	–	–	–
		`huge`	1.000	1.000	31.627	997.839	14.140	1.000	1.000	31.627	779.778	13.640
		`space`	1.000	0.927	17.330	2.419	823.680	1.000	0.917	18.827	2.570	719.690
		`QUIC`	1.000	1.000	4.794	150.143	1.910	1.000	1.000	8.040	253.418	2.053
Scale-free	500	`glasso`	0.966	1.000	4.806	16.135	0.110	1.000	1.000	4.857	70.186	0.940
		`clime`	0.992	1.000	18.027	389.842	630.516	1.000	1.000	5.360	41.360	429.616
		`GUEST`	0.989	1.000	4.582	5.154	591.700	0.989	1.000	4.712	5.239	467.888
		Wainwright	0.996	1.000	5.043	41.079	1.030	1.000	1.000	6.343	109.729	1.230
		`huge`	1.000	1.000	23.210	500.374	1.180	0.992	1.000	23.117	499.520	1.260
		`space`	0.981	0.982	23.670	9.015	192.000	0.981	0.924	34.589	8.983	213.640
		`QUIC`	1.000	1.000	5.109	82.918	0.330	1.000	1.000	7.229	137.900	0.350
	1000	`glasso`	1.000	1.000	6.185	58.995	8.330	0.992	1.000	7.404	33.694	10.550
		`clime`	–	–	–	–	–	–	–	–	–	–
		`GUEST`	0.988	0.998	5.589	5.800	858.570	0.984	0.997	6.112	6.961	1192.000
		Wainwright	1.000	1.000	7.550	203.058	9.400	1.000	0.992	18.934	464.820	14.660
		`huge`	1.000	1.000	32.229	1000.538	2.500	0.996	1.000	32.164	1000.239	3.570
		`space`	0.990	0.990	24.585	10.374	976.110	0.990	0.888	50.807	10.572	792.570
		`QUIC`	0.998	1.000	6.129	152.673	2.160	1.000	1.000	8.994	256.371	2.870

Table F2: Simulation results for the first scenario in Section F.1: graphical structure in Figure F1 under binary random variables

Graph	p	Methods	$\gamma = 0.85$					$\gamma = 0.9$				
			SPE	SEN	$\Vert\triangle_{\mathbf{\Theta}}\Vert_F$	LOSS_{KL}	Time	SPE	SEN	$\Vert\triangle_{\mathbf{\Theta}}\Vert_F$	LOSS_{KL}	Time
Lattice	500	`glasso`	0.948	1.000	78.642	1728.039	0.130	0.981	1.000	90.968	2000.734	0.110
		`clime`	1.000	0.000	140.131	2961.735	1680.000	1.000	0.000	166.147	3510.844	1685.800
		`GUEST`	0.944	0.913	7.074	5.875	523.860	0.948	0.846	6.323	5.450	763.950
		Wainwright	0.970	1.000	162.233	3581.826	0.190	0.993	1.000	209.631	4627.708	0.168
		`huge`	0.989	1.000	23.216	497.286	3.328	0.981	1.000	23.214	497.657	2.456
		`space`	0.974	0.971	8.644	6.093	251.010	0.970	0.996	6.516	10.366	228.006
		`QUIC`	0.936	1.000	60.064	1318.108	0.248	0.936	1.000	68.066	1496.180	0.334
	1000	`glasso`	0.969	1.000	111.222	3483.672	0.440	0.983	1.000	128.828	4035.316	0.150
		`clime`	–	–	–	–	–	–	–	–	–	–
		`GUEST`	0.969	0.955	49.562	11.258	1236.130	0.969	0.935	7.061	7.361	1481.400
		Wainwright	0.983	1.000	229.813	7218.377	0.728	0.981	1.000	296.316	9303.119	0.748
		`huge`	0.993	1.000	52.231	997.487	15.018	0.991	1.000	32.228	997.673	9.562
		`space`	0.983	1.000	56.010	14.853	947.400	0.992	1.000	8.081	10.022	649.738
		`QUIC`	0.967	1.000	84.935	2660.578	1.768	0.967	1.000	96.252	3017.405	2.054
Hub	500	`glasso`	0.969	1.000	78.215	1721.719	0.100	0.969	1.000	90.893	2001.530	0.170
		`clime`	1.000	0.000	139.076	2940.894	1685.920	1.000	0.000	165.687	3506.786	1704.080
		`GUEST`	0.969	0.844	5.065	4.818	791.350	0.969	0.879	4.520	4.996	754.390
		Wainwright	1.000	1.000	162.195	3583.674	0.182	1.000	1.000	209.616	1429.300	0.150
		`huge`	1.000	1.000	22.863	197.695	3.378	1.000	1.000	22.870	497.905	2.342
		`space`	0.996	0.940	9.033	6.010	182.886	1.000	0.946	8.492	5.722	232.452
		`QUIC`	0.962	1.000	59.947	1319.271	0.284	0.962	1.000	67.964	1497.348	0.312
	1000	`glasso`	0.986	1.000	111.684	3501.382	1.100	0.998	1.000	129.238	4051.221	1.210
		`clime`	–	–	–	–	–	–	–	–	–	–
		`GUEST`	0.982	0.980	7.459	5.166	2440.310	0.980	0.937	5.378	4.856	4130.070
		Wainwright	0.998	1.000	229.788	7219.973	0.906	1.000	1.000	296.305	9304.352	1.002
		`huge`	1.000	1.000	31.976	997.532	18.808	1.000	1.000	31.979	997.830	9.084
		`space`	0.998	0.990	8.543	5.286	633.936	1.000	1.000	6.497	5.442	854.548
		`QUIC`	0.980	1.000	84.852	2661.737	2.046	0.980	1.000	96.179	3018.564	1.916

Independence	500	`glasso`	1.000	1.000	78.840	1758.175	0.070	1.000	1.000	91.461	2038.349	0.080
		`clime`	1.000	0.997	135.573	2998.313	1130.890	1.000	0.995	162.503	3587.502	1082.600
		`GUEST`	1.000	0.997	3.732	0.702	455.140	1.000	1.000	0.979	0.200	425.720
		Wainwright	1.000	1.000	162.863	3636.151	0.166	1.000	1.000	209.856	4682.411	0.176
		`huge`	1.000	0.999	22.370	498.175	4.588	1.000	0.999	22.371	498.014	3.526
		`space`	1.000	0.999	4.453	1.311	241.000	1.000	0.990	4.049	1.001	304.000
		`QUIC`	1.000	1.000	60.075	1341.621	0.326	1.000	1.000	68.113	1521.085	0.334
	1000	`glasso`	1.000	1.000	111.317	3511.784	0.480	1.000	1.000	129.253	4074.778	0.330
		`clime`	–	–	–	–	–	–	–	–	–	–
		`GUEST`	1.000	0.999	3.245	0.429	1108.420	1.000	0.999	4.950	0.647	1239.060
		Wainwright	1.000	1.000	230.537	7280.676	0.976	1.000	1.000	297.159	9378.062	0.578
		`huge`	1.000	1.000	31.626	997.853	15.880	1.000	1.000	31.626	997.743	13.360
		`space`	1.000	0.998	4.651	0.600	959.684	1.000	0.998	6.385	0.945	606.142
		`QUIC`	1.000	1.000	84.984	2685.321	1.756	1.000	1.000	96.377	3045.153	1.334
Scale-free	500	`glasso`	0.969	1.000	78.407	1720.356	0.190	0.973	1.000	90.872	1995.070	0.110
		`clime`	1.000	0.910	139.221	2941.577	1821.860	0.996	0.988	163.469	3520.370	1415.970
		`GUEST`	0.958	0.961	6.465	8.388	584.880	0.966	0.970	7.213	7.058	390.640
		Wainwright	0.969	1.000	7770.507	3450.568	0.150	–	–	–	–	–
		`huge`	0.992	0.991	23.482	499.120	3.550	0.996	0.993	23.360	498.806	1.910
		`space`	0.969	0.898	23.887	9.489	786.640	0.985	0.829	24.572	10.170	182.960
		`QUIC`	0.958	1.000	59.893	1311.027	0.350	0.958	1.000	67.947	1489.075	0.200
	1000	`glasso`	0.996	1.000	128.481	4021.047	1.080	0.996	1.000	50.38	128.481	4021.047
		`clime`	–	–	–	–	–	–	–	–	–	–
		`GUEST`	0.978	0.994	15.572	8.422	2262.290	0.978	0.979	8.918	7.184	3141.670
		Wainwright	–	–	–	–	–	–	–	–	–	–
		`huge`	0.999	0.998	32.382	998.647	8.530	0.999	0.998	32.383	998.647	10.990
		`space`	0.996	0.466	32.328	10.194	852.830	0.990	0.982	15.793	8.386	881.800
		`QUIC`	0.978	1.000	96.056	3004.720	2.140	0.978	1.000	96.056	3004.720	1.760

Table F3: Simulation results for the first scenario in Section F.1: graphical structure in Figure F1 under count random variables

Graph	p	Methods	$(\lambda, \pi) = (0.5, 0.5)$					$(\lambda, \pi) = (0.8, 0.5)$				
			SPE	SEN	$\|\triangle_{\Theta}\|_F$	LOSS_{KL}	Time	SPE	SEN	$\|\triangle_{\Theta}\|_F$	LOSS_{KL}	Time
Lattice	500	glasso	1.000	0.743	9.481	15.016	0.220	1.000	0.745	9.439	15.740	0.530
		clime	1.000	0.000	10.088	68.496	767.060	1.000	0.000	10.073	67.832	831.490
		GUEST	0.963	0.994	5.026	4.130	36.640	0.978	1.000	4.835	3.971	63.230
		Wainwright	0.993	0.998	8.040	82.660	1.218	1.000	0.989	8.145	82.561	1.208
		huge	1.000	1.000	22.909	496.856	2.526	0.998	1.000	22.924	496.876	2.588
		space	1.000	1.000	6.455	8.620	234.472	1.000	1.000	7.597	14.039	236.542
		QUIC	1.000	1.000	7.078	48.582	0.990	1.000	1.000	7.075	48.563	0.892
	1000	glasso	1.000	0.776	14.453	117.293	5.000	1.000	0.775	14.410	118.043	4.000
		clime	–	–	–	–	–	–	–	–	–	–
		GUEST	0.992	1.000	4.356	4.124	369.500	0.994	1.000	4.634	5.065	464.360
		Wainwright	–	–	–	–	–	–	–	–	–	–
		huge	1.000	1.000	32.028	996.904	12.520	1.000	1.000	32.036	996.921	10.934
		space	1.000	0.999	5.626	4.293	704.566	1.000	0.999	5.498	14.278	697.000
		QUIC	1.000	1.000	7.240	84.472	6.716	1.000	1.000	7.237	84.369	7.812
Hub	500	glasso	1.000	0.748	8.577	16.548	0.410	1.000	0.746	8.598	16.121	0.470
		clime	1.000	0.000	9.097	64.954	1205.500	1.000	0.000	9.063	62.926	1119.190
		GUEST	0.985	1.000	3.120	3.025	78.880	0.985	1.000	3.826	3.875	50.630
		Wainwright	1.000	1.000	6.914	82.627	0.850	1.000	1.000	6.932	82.656	1.354
		huge	1.000	1.000	22.694	497.093	1.808	1.000	1.000	22.702	497.105	2.184
		space	1.000	1.000	3.575	3.739	226.956	1.000	1.000	5.560	10.729	232.916
		QUIC	1.000	1.000	5.866	48.613	0.662	1.000	1.000	5.873	48.601	0.762
	1000	glasso	1.000	0.776	13.893	118.322	3.200	1.000	0.777	13.935	120.828	4.360
		clime	–	–	–	–	–	–	–	–	–	–
		GUEST	0.998	1.000	4.079	4.447	396.350	0.996	1.000	3.666	3.863	465.640
		Wainwright	–	–	–	–	–	–	–	–	–	–
		huge	1.000	1.000	31.867	997.129	9.844	1.000	1.000	31.873	997.133	10.534
		space	1.000	1.000	4.092	5.840	973.352	1.000	1.000	3.770	10.819	963.304
		QUIC	1.000	1.000	6.069	85.123	5.650	1.000	1.000	6.074	85.023	5.264

Independence	500	glasso	1.000	1.000	1.891	21.391	0.200	1.000	1.000	2.029	18.677	0.340
		clime	1.000	1.000	5.388	100.447	823.520	1.000	1.000	5.408	95.672	833.850
		GUEST	1.000	1.000	0.038	0.010	51.240	1.000	1.000	0.028	0.009	53.960
		Wainwright	1.000	0.998	4.095	87.025	0.770	1.000	0.998	4.171	85.123	0.782
		huge	1.000	0.967	22.528	499.713	2.690	1.000	0.977	22.527	499.711	2.708
		space	1.000	0.985	6.982	1.207	412.366	1.000	0.992	4.910	0.960	310.932
		QUIC	1.000	0.998	2.136	44.414	0.200	1.000	0.998	2.355	45.921	0.322
	1000	glasso	1.000	1.000	4.442	112.268	3.470	1.000	1.000	4.468	119.455	3.470
		clime	–	–	–	–	–	–	–	–	–	–
		GUEST	1.000	1.000	0.029	0.008	118.120	1.000	1.000	0.458	0.195	441.600
		Wainwright	–	–	–	–	–	–	–	–	–	–
		huge	1.000	0.994	31.731	999.355	13.154	1.000	0.994	31.730	999.457	12.952
		space	1.000	0.881	18.863	2.585	702.156	1.000	0.886	18.322	2.561	700.488
		QUIC	1.000	0.998	2.801	82.402	1.458	1.000	1.000	2.962	83.791	2.106
Scale-free	500	glasso	1.000	0.947	8.125	14.655	1.440	1.000	0.947	8.129	14.734	1.280
		clime	0.996	0.929	9.381	72.293	1545.110	0.992	0.929	9.375	72.131	1529.860
		GUEST	0.969	0.995	6.134	3.830	125.960	0.973	0.995	5.990	3.908	116.810
		Wainwright	–	–	–	–	–	–	–	–	–	–
		huge	1.000	0.998	22.869	496.436	1.790	1.000	0.998	22.876	496.576	2.360
		space	0.992	0.982	16.944	4.718	251.130	0.992	0.983	16.053	5.047	256.210
		QUIC	1.000	0.993	6.308	50.020	0.860	1.000	0.993	6.311	49.885	0.840
	1000	glasso	1.000	0.953	12.604	119.365	8.630	1.000	0.953	12.604	119.365	7.980
		clime –	–	–	–	–	–	–	–	–	–	
		GUEST	0.992	0.997	4.059	5.905	433.030	0.992	1.000	3.820	4.304	419.050
		Wainwright	–	–	–	–	–	–	–	–	–	–
		huge	1.000	0.999	32.139	996.638	5.030	1.000	0.999	32.139	996.638	8.370
		space	1.000	0.921	40.242	8.006	746.270	1.000	0.921	40.242	8.006	715.110
		QUIC	1.000	0.994	6.897	84.730	4.270	1.000	0.994	6.897	84.730	5.260

Table F4: Simulation results for the second scenario in Section F.1: a *non-sparse* graphical structure

p	Methods	$\boldsymbol{\Sigma}_\epsilon = 0.1\mathbf{I}_p$				$\boldsymbol{\Sigma}_\epsilon = 0.3\mathbf{I}_p$			
		SPE	$\lVert\triangle_{\boldsymbol{\Theta}}\rVert_F$	LOSS_{KL}	Time	SPE	$\lVert\triangle_{\boldsymbol{\Theta}}\rVert_F$	LOSS_{KL}	Time
500	`glasso`	0.479	404.066	6214.581	122.286	0.452	401.826	4026.536	18.880
	`clime`	0.092	399.787	1537.956	2297.780	1.000	399.813	1243.193	1858.738
	`GUEST`	1.000	98.900	493.984	1318.162	1.000	98.901	493.984	514.298
	Wainwright	0.397	407.832	8739.234	27.446	0.282	409.692	9720.947	10.254
	`huge`	0.369	399.318	498.486	4.610	0.370	399.312	498.539	3.768
	`space`	1.000	446.590	1989.923	247.360	1.000	422.424	1990.831	195.500
	`QUIC`	0.550	402.486	4779.017	3.426	0.494	401.306	3324.283	2.776
	RLDA	1.000	307239.200	3813.786	0.132	1.000	308462.100	7559.651	0.140
1000	`glasso`	0.297	804.157	12560.790	341.528	0.285	801.870	8154.447	124.852
	`clime`	–	–	–	–	–	–	–	–
	`GUEST`	1.000	198.901	993.979	2551.170	1.000	198.901	993.979	2573.570
	Wainwright	0.237	808.076	17721.280	145.474	0.184	810.361	20054.420	99.128
	`huge`	0.221	799.314	997.814	11.606	0.223	799.305	997.885	9.120
	`space`	1.000	1877.299	3527.199	854.286	1.000	1585.678	3675.312	889.368
	`QUIC`	0.350	802.529	9641.092	26.430	0.308	801.331	6717.256	22.744
	RLDA	1.000	1230906.000	13674.850	0.686	1.000	1235861.000	27022.680	0.722

Table F5: Simulation results of classification for the first scenario in Section F.1 under the continuous random variables $\mathbf{X}$

Graph	p	Method	$\boldsymbol{\Sigma}_\epsilon = 0.1\ \mathbf{I}_p$			$\boldsymbol{\Sigma}_\epsilon = 0.3\ \mathbf{I}_p$		
			PRE	REC	F	PRE	REC	F
Lattice	500	glasso	0.847	0.727	0.783	0.889	0.793	0.838
		clime	0.848	0.788	0.817	0.841	0.780	0.809
		GUEST	0.868	0.879	0.873	0.890	0.852	0.871
		Wainwright	0.850	0.843	0.846	0.849	0.852	0.850
		huge	0.541	0.974	0.696	0.545	0.532	0.538
		space	0.786	0.780	0.783	0.776	0.760	0.768
		QUIC	0.838	0.847	0.842	0.852	0.852	0.852
	1000	glasso	0.950	0.940	0.945	0.933	0.914	0.923
		clime	–	–	–	–	–	–
		GUEST	0.947	0.947	0.947	0.933	0.918	0.925
		Wainwright	–	–	–	–	–	–
		huge	0.526	0.490	0.507	0.553	0.196	0.289
		space	0.922	0.922	0.922	0.919	0.904	0.912
		QUIC	0.938	0.927	0.932	0.939	0.900	0.919
Hub	500	glasso	0.815	0.821	0.818	0.873	0.816	0.843
		clime	0.811	0.818	0.814	0.878	0.792	0.833
		GUEST	0.832	0.825	0.829	0.876	0.811	0.843
		Wainwright	0.843	0.820	0.831	0.852	0.805	0.828
		huge	0.535	0.732	0.618	0.554	0.155	0.242
		space	0.777	0.747	0.762	0.806	0.737	0.770
		QUIC	0.829	0.820	0.825	0.862	0.797	0.828
	1000	glasso	0.936	0.936	0.936	0.926	0.911	0.918
		clime	–	–	–	–	–	–
		GUEST	0.940	0.936	0.938	0.929	0.911	0.920
		Wainwright	–	–	–	–	–	–
		huge	0.521	0.502	0.511	0.534	0.445	0.486
		space	0.902	0.899	0.901	0.872	0.883	0.877
		QUIC	0.933	0.907	0.920	0.917	0.895	0.906
Independence	500	glasso	0.829	0.836	0.834	0.864	0.833	0.848
		clime	0.820	0.837	0.828	0.891	0.804	0.845
		GUEST	0.844	0.854	0.849	0.854	0.865	0.859
		Wainwright	0.833	0.797	0.815	0.825	0.822	0.823
		huge	0.000	0.000	–	0.517	0.977	0.677
		space	0.803	0.806	0.804	0.799	0.818	0.808
		QUIC	0.832	0.793	0.812	0.816	0.826	0.821
	1000	glasso	0.911	0.914	0.913	0.900	0.914	0.907
		clime	–	–	–	–	–	–
		GUEST	0.923	0.937	0.930	0.903	0.915	0.909
		Wainwright	–	–	–	–	–	–
		huge	0.513	0.942	0.664	0.333	0.254	0.485
		space	0.878	0.891	0.885	0.883	0.911	0.897
		QUIC	0.913	0.930	0.921	0.894	0.890	0.892
Scale-free	500	glasso	0.843	0.862	0.852	0.848	0.827	0.837
		clime	0.811	0.889	0.848	0.843	0.819	0.831
		GUEST	0.838	0.870	0.853	0.839	0.865	0.852
		Wainwright	0.842	0.858	0.850	0.854	0.819	0.836
		huge	0.525	0.923	0.669	0.529	0.518	0.523
		space	0.790	0.808	0.799	0.811	0.723	0.717
		QUIC	0.824	0.867	0.845	0.852	0.831	0.841
	1000	glasso	0.943	0.936	0.939	0.919	0.923	0.921
		clime	–	–	–	–	–	–
		GUEST	0.956	0.956	0.956	0.930	0.933	0.931
		Wainwright	0.943	0.932	0.937	0.923	0.923	0.925
		huge	0.552	0.736	0.631	0.545	0.699	0.613
		space	0.875	0.894	0.884	0.777	0.795	0.786
		QUIC	0.943	0.932	0.937	0.923	0.931	0.927

Table F6: Simulation results of classification for the first scenario in Section F.1 under the binary random variables $\mathbf{X}$

Graph	p	Method	$\gamma = 0.85$			$\gamma = 0.90$		
			PRE	REC	F	PRE	REC	F
Lattice	500	glasso	0.867	0.884	0.875	0.850	0.844	0.847
		clime	0.864	0.864	0.864	0.840	0.820	0.830
		GUEST	0.860	0.919	0.889	0.853	0.883	0.868
		Wainwright	0.850	0.863	0.857	0.841	0.854	0.847
		huge	0.509	0.945	0.662	0.518	0.862	0.647
		space	0.835	0.832	0.834	0.830	0.826	0.828
		QUIC	0.847	0.887	0.866	0.849	0.819	0.834
	1000	glasso	0.881	0.866	0.873	0.922	0.882	0.869
		clime	–	–	–	–	–	–
		GUEST	0.974	0.823	0.892	0.962	0.851	0.903
		Wainwright	0.819	0.847	0.833	0.840	0.819	0.829
		huge	0.528	1.000	0.691	0.542	1.000	0.703
		space	0.850	0.820	0.835	0.890	0.834	0.861
		QUIC	0.822	0.836	0.829	0.825	0.856	0.840
Hub	500	glasso	0.823	0.862	0.842	0.837	0.827	0.832
		clime	0.828	0.854	0.840	0.822	0.806	0.814
		GUEST	0.808	0.881	0.843	0.840	0.859	0.849
		Wainwright	0.816	0.849	0.832	0.820	0.807	0.813
		huge	0.530	1.000	0.693	0.400	0.008	0.016
		space	0.820	0.842	0.831	0.813	0.802	0.807
		QUIC	0.835	0.830	0.840	0.829	0.835	0.832
	1000	glasso	0.902	0.909	0.905	0.901	0.922	0.911
		clime	–	–	–	–	–	–
		GUEST	0.908	0.913	0.910	0.902	0.938	0.920
		Wainwright	0.820	0.834	0.827	0.811	0.822	0.816
		huge	0.514	1.000	0.679	0.375	0.014	0.024
		space	0.830	0.830	0.830	0.846	0.830	0.838
		QUIC	0.825	0.814	0.820	0.831	0.834	0.833
Independence	500	glasso	0.823	0.816	0.819	0.831	0.841	0.836
		clime	0.815	0.808	0.811	0.837	0.837	0.837
		GUEST	0.837	0.824	0.831	0.821	0.857	0.838
		Wainwright	0.833	0.817	0.824	0.851	0.825	0.838
		huge	0.510	0.969	0.668	0.526	1.000	0.690
		space	0.831	0.825	0.828	0.831	0.833	0.832
		QUIC	0.803	0.840	0.821	0.831	0.844	0.838
	1000	glasso	0.917	0.928	0.923	0.900	0.900	0.900
		clime	–	–	–	–	–	–
		GUEST	0.911	0.936	0.923	0.918	0.940	0.929
		Wainwright	0.908	0.915	0.912	0.917	0.902	0.910
		huge	0.467	0.460	0.463	0.000	0.000	–
		space	0.927	0.915	0.921	0.900	0.935	0.922
		QUIC	0.913	0.891	0.902	0.844	0.831	0.837
Scale-free	500	glasso	0.848	0.820	0.833	0.818	0.808	0.813
		clime	0.872	0.812	0.841	0.811	0.808	0.810
		GUEST	0.882	0.888	0.885	0.846	0.852	0.849
		Wainwright	0.510	0.510	0.510	–	–	–
		huge	0.540	0.847	0.660	0.497	0.367	0.423
		space	0.757	0.751	0.754	0.755	0.743	0.749
		QUIC	0.828	0.885	0.856	0.812	0.812	0.812
	1000	glasso	0.900	0.907	0.903	0.834	0.907	0.920
		clime	–	–	–	–	–	–
		GUEST	0.929	0.925	0.927	0.939	0.927	0.933
		Wainwright	–	–	–	–	–	–
		huge	0.545	0.219	0.312	0.572	0.448	0.502
		space	0.861	0.830	0.845	0.824	0.815	0.819
		QUIC	0.904	0.911	0.907	0.922	0.907	0.915

Table F7: Simulation results of classification for the first scenario in Section F.1 under the count random variables $\mathbf{X}$

Graph	p	Method	$(\lambda,\pi)=(0.5,0.5)$			$(\lambda,\pi)=(0.8,0.5)$		
			PRE	REC	F	PRE	REC	F
Lattice	500	glasso	0.557	0.529	0.543	0.576	0.529	0.552
		clime	0.591	0.580	0.585	0.564	0.533	0.548
		GUEST	0.829	0.802	0.815	0.871	0.833	0.851
		Wainwright	0.634	0.604	0.618	0.616	0.595	0.605
		huge	0.516	0.451	0.481	0.519	0.471	0.494
		space	0.556	0.529	0.542	0.562	0.541	0.551
		QUIC	0.607	0.588	0.598	0.608	0.598	0.618
	1000	glasso	0.578	0.576	0.577	0.577	0.564	0.570
		clime	–	–	–	–	–	–
		GUEST	0.832	0.821	0.827	0.877	0.891	0.884
		Wainwright	–	–	–	–	–	–
		huge	0.516	0.549	0.532	0.486	0.429	0.456
		space	0.566	0.568	0.567	0.552	0.544	0.548
		QUIC	0.630	0.603	0.616	0.619	0.599	0.609
Hub	500	glasso	0.576	0.591	0.583	0.610	0.527	0.566
		clime	0.582	0.595	0.588	0.578	0.546	0.561
		GUEST	0.853	0.864	0.859	0.893	0.827	0.858
		Wainwright	0.574	0.583	0.578	0.539	0.596	0.566
		huge	0.509	0.587	0.545	0.479	0.535	0.505
		space	0.533	0.543	0.538	0.485	0.552	0.516
		QUIC	0.575	0.587	0.581	0.540	0.591	0.564
	1000	glasso	0.597	0.549	0.572	0.593	0.566	0.579
		clime	–	–	–	–	–	–
		GUEST	0.840	0.825	0.833	0.869	0.843	0.856
		Wainwright	–	–	–	–	–	–
		huge	0.529	0.537	0.533	0.548	0.560	0.554
		space	0.551	0.544	0.548	0.568	0.515	0.540
		QUIC	0.582	0.575	0.579	0.583	0.553	0.568
Independence	500	glasso	0.876	0.840	0.858	0.801	0.850	0.825
		clime	0.872	0.840	0.856	0.830	0.865	0.847
		GUEST	0.879	0.836	0.858	0.820	0.877	0.848
		Wainwright	0.769	0.945	0.848	0.867	0.791	0.827
		huge	0.544	1.000	0.705	0.333	0.004	0.008
		space	0.749	0.938	0.832	0.806	0.799	0.802
		QUIC	0.746	0.960	0.839	0.881	0.774	0.824
	1000	glasso	0.925	0.874	0.899	0.898	0.935	0.916
		clime	–	–	–	–	–	–
		GUEST	0.929	0.874	0.901	0.894	0.958	0.925
		Wainwright	–	–	–	–	–	–
		huge	0.466	1.000	0.636	0.431	0.090	0.149
		space	0.932	0.820	0.872	0.920	0.844	0.880
		QUIC	0.948	0.854	0.898	0.924	0.902	0.913
Scale-free	500	glasso	0.540	0.535	0.538	0.581	0.593	0.587
		clime	0.562	0.548	0.555	0.578	0.581	0.579
		GUEST	0.815	0.864	0.839	0.839	0.885	0.861
		Wainwright	–	–	–	–	–	–
		huge	0.492	0.506	0.499	0.457	0.534	0.492
		space	0.529	0.564	0.546	0.546	0.581	0.563
		QUIC	0.548	0.544	0.546	0.583	0.593	0.588
	1000	glasso	0.589	0.575	0.582	0.565	0.565	0.565
		clime	–	–	–	–	–	–
		GUEST	0.882	0.802	0.892	0.849	0.844	0.847
		Wainwright	–	–	–	–	–	–
		huge	0.529	0.567	0.548	0.540	0.515	0.527
		space	0.584	0.602	0.593	0.547	0.544	0.545
		QUIC	0.596	0.575	0.585	0.572	0.570	0.571

Table F8: Simulation results of classification for the second scenario in Section F.1

p	Method	$\boldsymbol{\Sigma}_\epsilon = 0.1\ \mathbf{I}_p$			$\boldsymbol{\Sigma}_\epsilon = 0.3\ \mathbf{I}_p$		
		PRE	REC	F	PRE	REC	F
500	`glasso`	0.842	0.848	0.845	0.854	0.871	0.863
	`clime`	0.549	0.527	0.538	0.573	0.551	0.562
	`GUEST`	0.945	0.923	0.934	0.945	0.896	0.920
	Wainwright	0.842	0.848	0.845	0.852	0.852	0.852
	`huge`	0.545	0.523	0.534	0.545	0.496	0.519
	`space`	0.559	0.542	0.550	0.549	0.523	0.536
	`QUIC`	0.839	0.848	0.844	0.855	0.875	0.865
	RLDA	0.545	0.523	0.534	0.545	0.496	0.519
1000	`glasso`	0.926	0.923	0.924	0.902	0.913	0.907
	`clime`	–	–	–	–	–	–
	`GUEST`	0.950	0.909	0.929	0.938	0.932	0.935
	Wainwright	0.925	0.915	0.920	0.895	0.913	0.904
	`huge`	0.551	0.517	0.533	0.536	0.532	0.534
	`space`	0.550	0.509	0.529	0.524	0.520	0.522
	`QUIC`	0.926	0.926	0.926	0.902	0.913	0.907
	RLDA	0.550	0.509	0.529	0.538	0.532	0.535
